# Genome‐Wide Codon Reprogramming Enables a Multifactorially Attenuated Influenza Vaccine with Broad Cross‐Protection

**DOI:** 10.1002/advs.202516448

**Published:** 2025-11-30

**Authors:** Yang Wang, Tianxin Ma, Yujiao He, Qinming Li, Kailin Mai, Minying Mo, Chenyang Cao, Jiahui Li, Pei Feng, Jiaojiao Peng, Jing Sun, Weiqi Pan, Zifeng Yang, Ling Chen

**Affiliations:** ^1^ State Key Laboratory of Respiratory Disease National Clinical Research Center for Respiratory Disease National Center for Respiratory Medicine Guangzhou Institute of Respiratory Health the First Affiliated Hospital of Guangzhou Medical University Guangzhou 510370 China; ^2^ Guangzhou National Laboratory Guangzhou 510005 China; ^3^ Guangdong Laboratory of Computational Biomedicine Guangzhou Institutes of Biomedicine and Health Chinese Academy of Sciences Guangzhou 510530 China; ^4^ Respiratory Disease AI Laboratory on Epidemic and Medical Big Data Instrument Applications Faculty of Innovation Engineering Macau University of Science and Technology Macau SAR 999078 China; ^5^ Guangdong Cardiovascular Institute Medical Research Institute Guangdong Provincial People's Hospital (Guangdong Academy of Medical Sciences) Southern Medical University Guangzhou 510080 China; ^6^ Institute of Biochemistry & Molecular Biology School of Basic Medical Sciences Guangdong Medical University Dongguan 523808 China

**Keywords:** cross‐protective immunity, host antiviral restriction factors, influenza A virus, live attenuated vaccines, synonymous codon reprogramming

## Abstract

Live attenuated influenza vaccines (LAIVs) can elicit broad immunity, but rational attenuation strategies are limited. PR8^rp^, a prototype influenza A virus with five segments extensively reprogrammed to use the least‐preferred synonymous codons is generated, introducing 1956 silent mutations and elevating CpG content. PR8^rp^ exhibits profound attenuation in vitro and ≈20 000‐fold lower virulence in mice, yet maintains vaccine‐level yields. A single intranasal dose confers sterilizing homologous protection and dose‐dependent cross‐protection against heterologous H1N1pdm and heterosubtypic H3N2 challenge, mediated by homologous neutralizing antibodies, cross‐reactive non‐neutralizing antibodies, and IFN‐γ–biased T cell responses. Mechanistic analyses reveal that attenuation resulted from defective NA genome packaging, loss of NS1 protein accumulation, augment of host antiviral responses, and heightened susceptibility to zinc‐finger antiviral protein–mediated restriction, rather than impaired RNA or protein synthesis. Applying this approach to a contemporary H1N1 strain yielded similar stable attenuation. These findings establish genome‐wide codon reprogramming as a versatile platform for safe, broadly protective LAIVs with multiple attenuation mechanisms.

## Graphical abstract

Genome‐wide codon reprogramming of influenza A virus introduces 1956 synonymous mutations across five segments and elevates CpG content, causing defective NA packaging, loss of NS1 accumulation, and enhanced ZAP‐mediated antiviral responses without affecting RNA or protein synthesis. These mechanisms result in profound attenuation and potent homologous and heterologous protection, establishing a versatile platform for safe, broadly protective LAIVs.

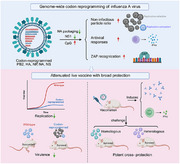

## 1 Introduction

Seasonal influenza viruses cause approximately one billion infections each year and result in 290 000 to 650 000 deaths worldwide.^[^ ^1^ ^]^ Although the COVID‐19 pandemic and related nonpharmaceutical interventions temporarily suppressed influenza circulation, the virus has resurged following the relaxation of these measures.^[^ ^2^ ^]^ Influenza A and B viruses—members of the Orthomyxoviridae family—are enveloped, negative‐sense RNA viruses with genomes segmented into eight distinct RNA segments.^[^ ^3^ ^]^ Each segment typically encodes one or more proteins essential for the viral life cycle: polymerase basic protein 2 (PB2, segment 1), polymerase basic protein 1 (PB1, segment 2), polymerase acidic protein (PA, segment 3), hemagglutinin (HA; segment 4), nucleoprotein (NP; segment 5), neuraminidase (NA; segment 6), matrix proteins M1 and M2 (segment 7), and the nonstructural proteins NS1 and NS2/NEP (segment 8).^[^ ^4^ ^]^

Vaccination remains the primary defense against influenza; however, the effectiveness of traditional inactivated vaccines remains moderate (≈19–60%), largely due to continual antigenic drift and limited durability of protection.^[^ ^4^, ^5^, ^6^ ^]^ Live attenuated influenza vaccines better mimic natural infection and induce mucosal and cellular immunity.^[^ ^7^, ^8^, ^9^ ^]^ Nevertheless, concerns regarding safety, genetic stability, and antigenic match have restricted their broader use.

A promising alternative is synonymous genome reprogramming, in which viral coding sequences are extensively redesigned—modifying codons, codon pairs, or dinucleotide content (e.g., CpG and UpA)—without altering the encoded amino acids.^[^ ^10^, ^11^, ^12^, ^13^, ^14^, ^15^, ^16^, ^17^ ^]^ This strategy preserves all native antigenic epitopes while suppressing viral fitness. Reprogramming can take various algorithms,^[^ ^18^ ^]^ including using codons^[^ ^11^ ^]^ or codon pairs^[^ ^10^, ^12^, ^17^ ^]^ that are infrequently used in mammals, preferred by avian influenza genomes,^[^ ^13^ ^]^ prone to mutate into stop codons,^[^ ^14^ ^]^ or even those commonly used in mammals.^[^ ^16^ ^]^ These diverse reprogramming strategies impair viral fitness through several intertwined mechanisms: slowing translation efficiency and destabilizing mRNA;^[^ ^19^ ^]^ increasing CpG/UpA dinucleotide frequencies, which heighten detection by innate immune factors such as the zinc‐finger antiviral protein (ZAP) and RNase L^[^ ^20^, ^21^, ^22^ ^]^ and stimulate interferon responses;^[^ ^23^, ^24^ ^]^ and disrupting genome packaging efficiency.^[^ ^16^ ^]^

A key advantage of large‐scale synonymous reprogramming lies in its inherent safety: by introducing hundreds of silent mutations across the viral genome, the probability of simultaneous reversion to a virulent phenotype is extraordinarily low.^[^ ^10^, ^25^ ^]^ Moreover, the level of attenuation can be tuned by the extent of recoding, with greater numbers of synonymous changes yielding stronger attenuation.^[^ ^17^ ^]^ Increasing the mutation load amplifies the cumulative effects of recoding, further slowing translation, increasing RNA instability, and enhancing ZAP‐mediated recognition.^[^ ^19^, ^20^ ^]^

In this study, we sought to maximize the extent of synonymous genome reprogramming in the influenza virus by introducing the largest feasible number of silent mutations. To achieve this, we developed a novel recoding strategy that replaces viral coding sequences with codons rarely used by influenza viruses and applied it to as many gene segments as possible. Using this approach, we generated a recombinant virus in which the PB2, HA, NP, NA, and NS gene segments were extensively recoded with these rarely used codons. We then comprehensively evaluated its replication kinetics, virulence, and protective efficacy against both homologous and heterologous challenges. Furthermore, we investigated the mechanisms underlying its attenuation, focusing on viral genome packaging, RNA and protein synthesis, host innate immune responses, and recognition by the ZAP.

## 2 Results

### 2.1 Successful Rescue of an Influenza A Virus Containing Five Extensively Codon‐Reprogrammed Segments

We reprogrammed the A/Puerto Rico/8/34 (PR8) genome to use the least frequently employed synonymous codons in influenza A virus (Table S1, Supporting Information), while preserving the known packaging signals at the 3′ and 5′ termini of each segment, except for the NA segment, in which only the minimal required packaging signal was retained to deliberately impair its packaging efficiency and enhance attenuation.^[^ ^16^ ^]^ For NS and M, only part of the NS1 and M1 coding sequences were reprogrammed to avoid altering downstream NS2 and M2 reading frames.

**Figure 1:**
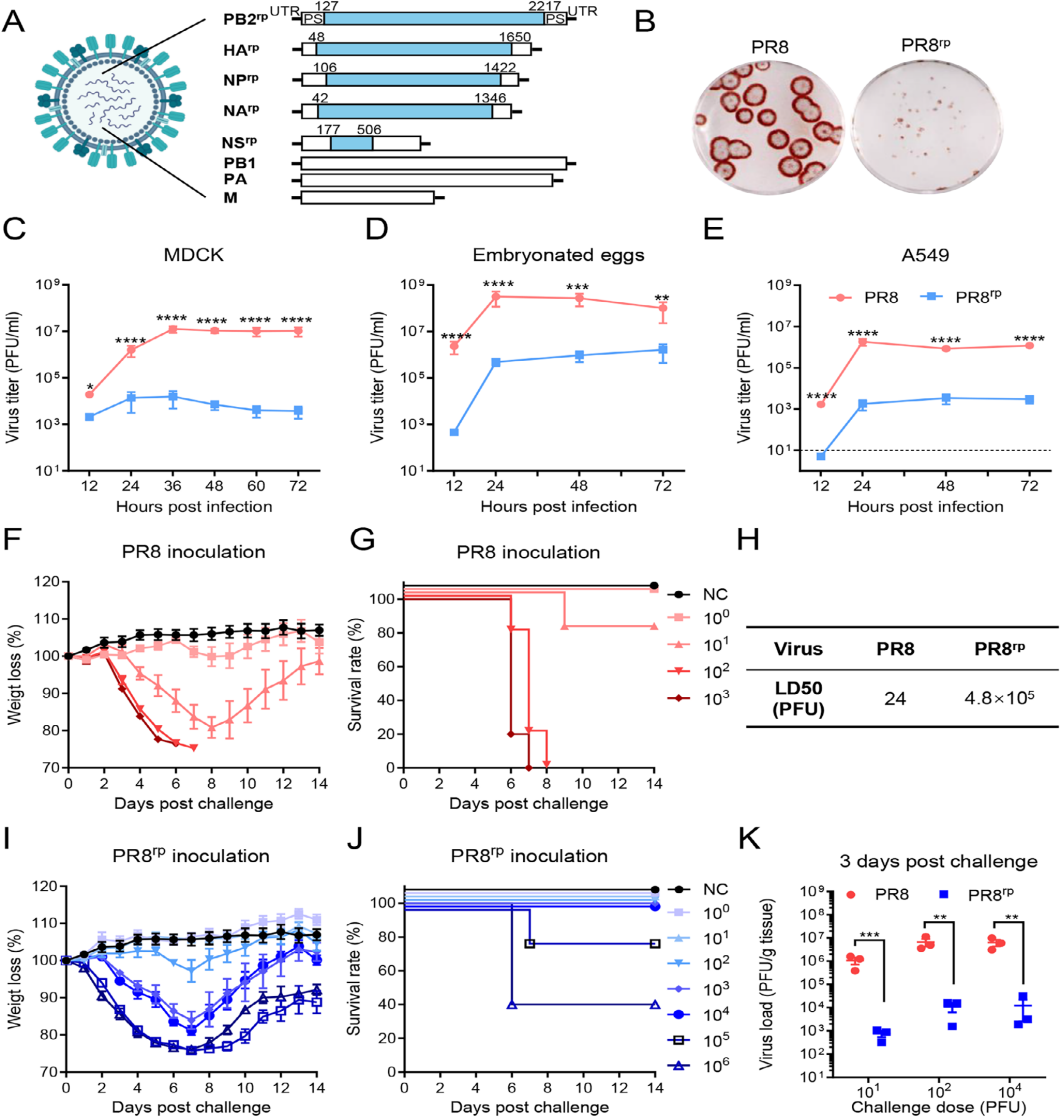
Construction and characterization of the codon‐reprogrammed influenza virus PR8^rp^. A) Schematic of PR8^rp^ genome design. The virus contains wild‐type PB1, PA, and M segments from PR8, and extensively reprogrammed PB2, HA, NP, NA, and NS segments (PB2^rp^, HA^rp^, NP^rp^, NA^rp^, NS^rp^) using influenza virus's least‐preferred synonymous codons, retaining original 5′ and 3′ packaging signals (PS). B) Plaque morphology of PR8 and PR8^rp^ viruses in MDCK cells. C–E) Multi‐step growth curves of PR8 (red circles) and PR8^rp^ (blue squares) in MDCK cells (MOI = 0.01) (C), embryonated chicken eggs (100 plaque‐forming units (PFU) egg^−1^) (D), and A549 cells (multiplicity of infection (MOI) = 0.01) (E). The dashed line represents the detection limit. Each experiment was performed with three independent biological replicates. F,G) Body weight loss (F) and survival rates (G) of BALB/c mice (*n* = 5) after intranasal inoculation with the indicated doses of PR8 or PBS as a negative control (NC). H) LD_50_ values of PR8 and PR8^rp^ calculated by the Reed–Muench method based on data from (F,G) and (I,J). I,J) Body weight loss (I) and survival rates (J) of BALB/c mice (*n* = 5) after intranasal inoculation with the indicated doses of PR8^rp^ or PBS (NC). K) Lung viral titers in mice (*n* = 3) 3 days post‐infection (dpi) with PR8 (red circles) or PR8^rp^ (blue squares) at indicated doses. Viral titers were determined by plaque assays on MDCK cells. Data represent mean ± SEM. For comparisons between two groups with equal variances, unpaired two‐tailed Student's *t*‐tests were used. For analysis of growth curves, two‐way ANOVA with Šidák's multiple‐comparison correction was used to compare group means at each time point. *, *p* < 0.05; **, *p* < 0.01; ***, *p* < 0.001; ****, *p* < 0.0001.

Using reverse genetics, we first incorporated each reprogrammed segment individually into a wild‐type PR8 background to create 7 + 1 viruses. All single‐segment reprogrammed viruses were successfully rescued, confirming the compatibility of these recoded segments. Two‐ and three‐segment combinations (28 and 56 possible combinations, respectively) were also fully rescuable, whereas only a few four‐segment combinations remained viable. Importantly, we succeeded in rescuing a single five‐segment combination (PB2^rp,^ HA^rp^, NP^rp^, NA^rp^, NS^rp^), hereafter designated PR8^rp^ (**Figure** **1**A). All other five‐ or six‐segment combinations failed to yield infectious virus.

The complete genome of PB2^rp^ was verified by Sanger sequencing, confirming successful incorporation of all designed synonymous mutations without off‐target changes in each reprogrammed segment (Table S2, Supporting Information). PR8^rp^ harbors 1956 silent mutations across its 13 583 nt genome (14.4% overall mutation rate), with a gain of 772 CpG dinucleotides (**Table** **1**). Codon adaptation index (CAI) values dropped markedly relative to influenza codon usage but changed minimally relative to human codon usage.

**Table 1:** Characteristics of reprogrammed influenza genome segments.

Gene segment	Type	Substitutions	CAI		C+G%	No. CpGs	No. UpAs
			Relative to Homo sapiens^a)^	Relative to influenza A virus^b)^			
PB2	Wildtype	‐	0.731	0.81	43.40	49	108
	Reprogrammed	622	0.623	0.478	55.10	295	114
HA	Wildtype	‐	0.748	0.815	41.46	30	92
	Reprogrammed	455	0.661	0.496	53.01	204	94
NP	Wildtype	‐	0.749	0.798	46.13	43	52
	Reprogrammed	421	0.617	0.465	56.29	207	75
NA	Wildtype	‐	0.728	0.777	42.82	30	82
	Reprogrammed	377	0.631	0.485	56.48	184	48
NS	Wildtype	‐	0.709	0.783	44.72	24	34
	Reprogrammed	81	0.66	0.627	46.85	54	39
Total	Wildtype	‐	0.736	0.8	43.55	176	368
	Reprogrammed	1956	0.635	0.488	54.2	944	370

^a)^ The codon adaptation indexes were calculated to quantify the similarities in codon usage between them and the Homo sapiens genome reference by using CAIcal (https://ppuigbo.me/programs/CAIcal/);

^b)^ The codon adaptation indexes were calculated to quantify the similarities in codon usage between them and the Influenza A virus genome reference.

### 2.2 Codon Reprogramming Causes Strong Attenuation In Vitro and In Vivo

To determine whether extensive synonymous reprogramming impaired viral replication, we compared the growth of PR8^rp^ with wild‐type PR8 in cell cultures and eggs. PR8^rp^ formed remarkable smaller plaques than PR8 in MDCK cells (Figure 1B) and exhibited 10^2^–10^3^ reductions in endpoint titers across MDCK cells, A549 cells, and embryonated eggs (*p* = 0.0048–*p* < 0.0001; Figure 1C–E). Despite reduced growth in eggs, titers reached 1.6 × 10^6^ PFU mL^−1^, sufficient for live vaccine manufacturing.

We next examined whether the strong in vitro attenuation translated to reduced virulence in vivo. In BALB/c mice, PR8^rp^ showed ≈20 000‐fold attenuation in LD_50_ compared to PR8 (Figure 1F–J), with minimal weight loss or clinical symptoms even at doses of 10^2^–10^3^ PFU, and markedly reduced lung titers at 3 days post‐infection (dpi) (Figure 1K). These results confirmed that PR8^rp^ is highly attenuated in both cell cultures and animal models while retaining sufficient replication for manufacturing.

### 2.3 PR8^rp^ Vaccination Provides Potent Homologous and Heterologous Protection

**Figure 2:**
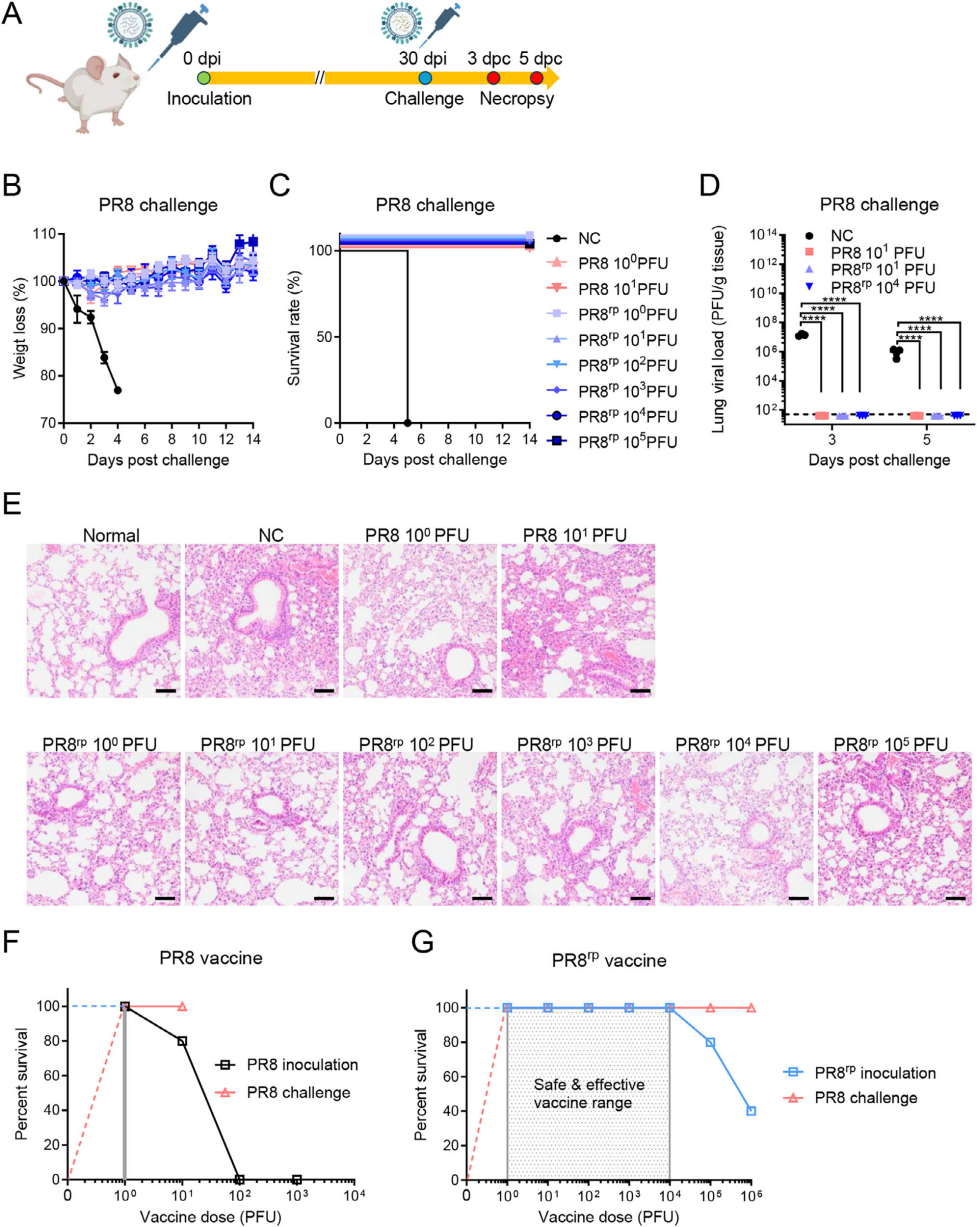
Protective efficacy of PR8^rp^ vaccination against homologous PR8 challenge in BALB/c mice. A) Experimental design. BALB/c mice were intranasally inoculated with varying doses of PR8, PR8^rp^, or an equal volume of PBS as an unvaccinated negative control (NC). Thirty days later, mice were challenged with 1000 × LD_50_ of homologous PR8. Body weight and survival were monitored for 14 days; lungs were collected on days 3 and 5 post‐challenge for viral titration and histopathology. B,C) Body weight changes (B) and survival rates (C) of vaccinated mice (*n* = 5 per group) following PR8 challenge. D) Lung viral titers at 3 and 5 dpc, determined by plaque assay on MDCK cells (*n* = 3 per group). E) Representative H&E staining of lung tissues collected at 5 dpc from normal mice, PBS controls (NC), and mice vaccinated with indicated doses of PR8 or PR8^rp^. Scale bars, 25 µm. F,G) Vaccine safety and effective dose ranges (shaded) for PR8 (F) and PR8^rp^ (G), showing doses that were non‐lethal upon inoculation and provided complete protection against lethal PR8 challenge. Data are shown as mean ± SEM. *p*‐values were calculated using one‐way analysis of variance (ANOVA) followed by Tukey's multiple‐comparison post hoc test. ****p < 0.0001.

Infection by a sub‐lethal dose of a virus can, in principle, provoke protective immunity, which is commonly observed in natural human infections. To evaluate the protective efficacy of PR8^rp^ as a live attenuated vaccine, BALB/c mice were intranasally immunized with sub‐lethal doses of PR8, PR8^rp^, or PBS as a control, and challenged 30 days later with 1000 × LD_50_ of the homologous PR8 virus (**Figure** **2**A). While all control mice succumbed, both PR8‐ and PR8^rp^‐vaccinated mice showed complete survival even at the lowest immunization dose of 1 PFU, with no measurable body weight loss (Figure 2B,C). Lung viral titers were undetectable at 3 and 5 days post‐challenge (dpc), confirming sterilizing immunity (Figure 2D), and histopathology revealed minimal pulmonary lesions in vaccinated mice compared to severe inflammation in controls (Figure 2E). PR8^rp^ showed a substantially wide safety margin (LD_50_/PD_50_ > 900 000) compared to PR8 (LD_50_/PD_50 _= 48) (Figure 2F,G), indicating that it confers potent homologous protection while maintaining a substantially improved safety profile.

**Figure 3:**
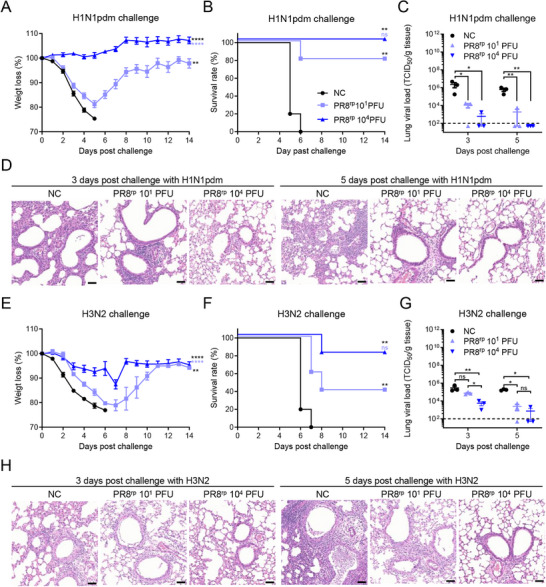
Protective efficacy of PR8^rp^ vaccination against heterologous challenges in BALB/c mice. BALB/c mice were intranasally inoculated with PR8^rp^ at the indicated doses or with PBS as a negative control (NC) and challenged 30 days later with heterologous influenza viruses. A–D) Challenge with A/California/04/2009 (H1N1pdm) at 10 × LD_50_: body weight changes (A, *n* = 5 per group), survival rates (B, *n* = 5 per group), lung viral loads at 3 and 5 dpc (C, *n* = 3 per group), and representative hematoxylin and eosin (H&E)‐stained lung sections (D). E–H) Challenge with A/Aichi/2/1968 (H3N2) at 3 × LD_50_: body weight changes (E, *n* = 5 per group), survival rates (F, *n* = 5 per group), lung viral loads at 3 and 5 dpc (G, *n *= 3 per group), and representative H&E‐stained lung sections (H). Viral titers in lung homogenates were determined by TCID_50_ assay on MDCK cells. Dashed lines in C and G indicate the limit of detection. Error bars represent SEM. For lung viral titers, one‐way ANOVA followed by Tukey's multiple‐comparison post hoc test was applied. For analysis of body weight changes over time, two‐way repeated‐measures ANOVA with Tukey's multiple‐comparison post hoc test was performed to evaluate the main effects among groups. Survival curves were analyzed using the log‐rank (Mantel–Cox) test. In the analyses of body weight loss and survival rates, black asterisks denote statistical significance compared with the NC group, while light blue asterisks denote significance compared with the PR8^rp^ 10^1^ PFU group. *, *p *< 0.05; **, *p* < 0.01; ns, not significant. Scale bars, 50 µm.

To further assess whether PR8^rp^ confers cross‐protection beyond the homologous strain, we evaluated its efficacy against heterologous and heterosubtypic influenza viruses. BALB/c mice were intranasally immunized with either a low dose (10 PFU) or high dose (10^4^ PFU) of PR8^rp^, or PBS as an unvaccinated control, and then challenged 30 days later with 10 × LD_50_ of the heterologous A/California/04/2009 (H1N1pdm) (**Figure** **3**A–D) or 3 × LD_50_ of the heterosubtypic A/Aichi/2/1968 (H3N2) virus (Figure 3E–H). Following the H1N1pdm challenge, all mice in the control group exhibited rapid and severe body weight loss beginning on day 1 post‐challenge and succumbed to infection by day 6 (Figure 3A,B). In contrast, PR8^rp^‐vaccinated mice displayed a clear dose‐dependent protective effect: those receiving the high dose were completely protected from mortality and showed only mild, transient weight loss (*p* < 0.0001), while the low‐dose group had partial protection with reduced body weight loss (*p* = 0.0013). Consistent with the clinical observations, lung viral titers at 3 and 5 dpc were significantly lower in both vaccinated groups compared to controls, with a 501‐ to 9840‐fold reduction (*p* = 0.0489–0.0012) (Figure 3C). Histopathological analysis further demonstrated that PR8^rp^ vaccination markedly reduced pulmonary inflammation and preserved alveolar structure compared to the severe bronchopneumonia seen in control mice (Figure 3D).

A similar pattern was observed against the H3N2 challenge (Figure 3E–H). Control mice experienced rapid weight loss and mortality, whereas PR8^rp^ vaccination significantly mitigated disease severity and improved survival in a dose‐dependent manner. High‐dose vaccination conferred near‐complete protection, characterized by minimal weight loss (*p* < 0.0001), substantially reduced lung viral titers (*p* = 0.0113–0.0020), and attenuated lung pathology compared with the low‐dose and control groups.

Collectively, these findings demonstrate that PR8^rp^ not only provides sterilizing immunity against homologous challenge but also confers strong, dose‐dependent protection against heterologous and heterosubtypic influenza virus infections, substantially reducing viral replication and pathology in the respiratory tract.

### 2.4 Humoral and Cellular Immune Responses Mediate Homologous and Heterologous Protection

**Figure 4:**
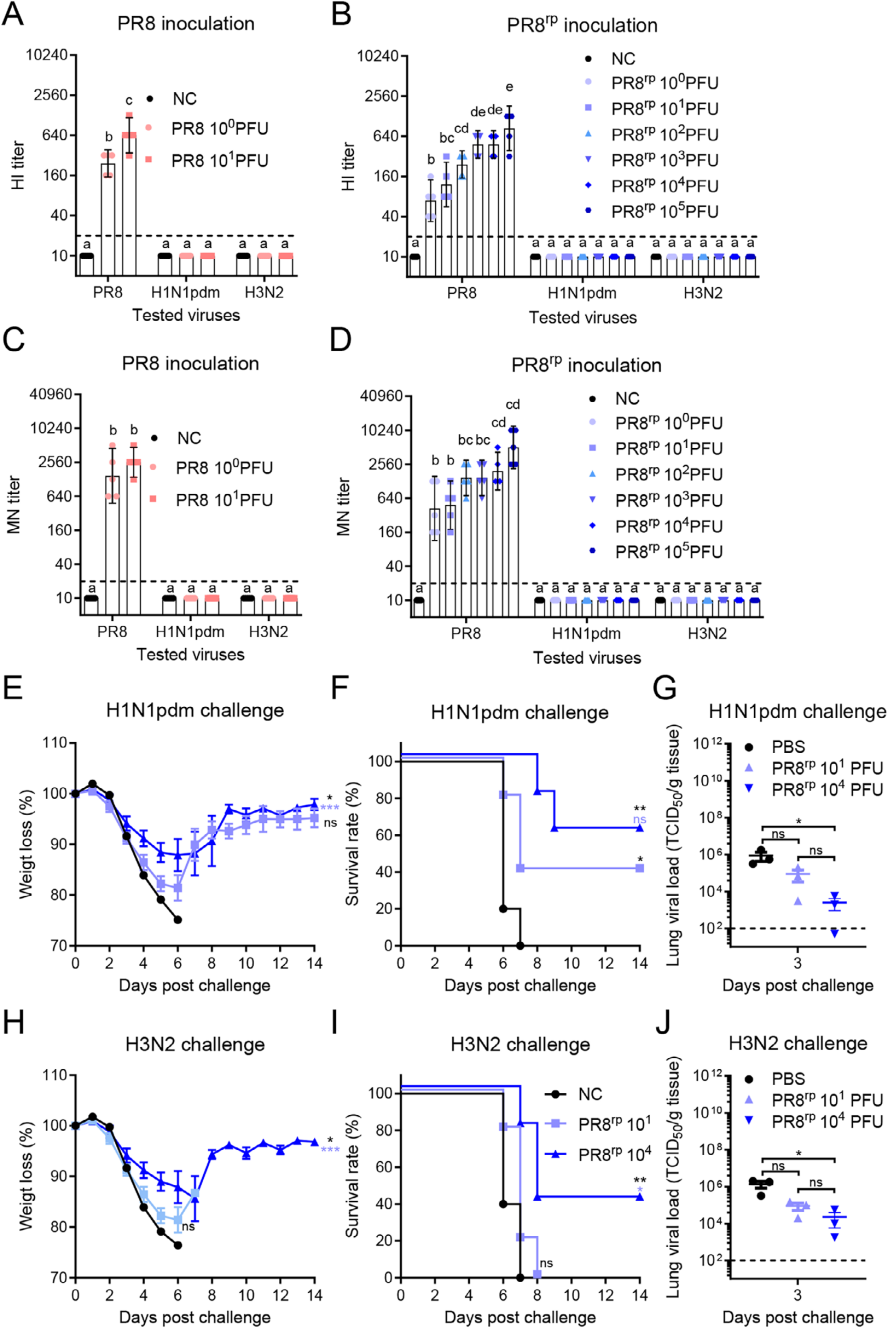
PR8^rp^‐induced robust humoral immunity contributes to homologous and heterologous protection. BALB/c mice were intranasally inoculated with various doses of PR8 (A,C) or PR8^rp^ (B,D) viruses, or PBS as an unvaccinated control (NC). Four weeks post‐vaccination, sera were collected and analyzed by hemagglutination inhibition (HI) (A,B) and microneutralization (MN) (C,D) assays against the homologous PR8 virus and heterologous A/California/04/2009 (H1N1pdm) and A/Aichi/2/1968 (H3N2) viruses. Data are shown as geometric means ± 95% CI (*n* = 5), with groups not sharing the same lowercase letter differing significantly (*p* < 0.05). For passive transfer experiments, pooled sera from mice vaccinated with PR8^rp^ (10^1^ or 10^4^ PFU) or from the NC group were administered intraperitoneally (200 µL) to naïve recipients 2 h before intranasal challenge with either 10 × LD_50_ H1N1pdm (E–G) or 3 × LD_50_ H3N2 (H–J). Body weight (E,H; *n* = 5 per group), survival rates (F,I; *n* = 5 per group), and lung viral titers at 3 days post‐challenge (G,J; *n* = 3 per group) were determined. Data are shown as mean ± SEM. For lung viral titers, one‐way ANOVA followed by Tukey's multiple‐comparison post hoc test was applied. For analysis of body weight changes, two‐way repeated‐measures ANOVA with Tukey's multiple‐comparison post hoc test was performed to evaluate the main effects among groups. Survival curves were analyzed using the log‐rank (Mantel–Cox) test. In the analyses of body weight loss and survival rates, black asterisks denote statistical significance compared with the NC group, while light blue asterisks denote significance compared with the PR8^rp^ 10^1^ PFU group. *, *p* < 0.05; **, *p* < 0.01; ***, *p* < 0.001; ns, not significant.

To better understand the immune responses associated with the protection observed in homologous and heterologous challenge experiments, we assessed both humoral and cellular responses following PR8^rp^ immunization. Four weeks post‐vaccination, functional antibody levels were measured by hemagglutination inhibition (HI) and microneutralization (MN) assays. Compared to the unvaccinated control group, mice intranasally inoculated with PR8^rp^ developed dose‐dependent HI and MN antibody titers against the homologous PR8 virus, with the highest titers observed at doses of 10^3^–10^5^ PFU (**Figure** **4**A–D). Neither PR8 nor PR8^rp^ induced detectable HI or MN antibodies against the heterologous H1N1pdm or H3N2 viruses.

To further evaluate the potential contribution of humoral immune responses to cross‐protection, passive transfer experiments were performed. Sera from PR8^rp^‐vaccinated mice (10^1^ or 10^4^ PFU) were administered to naïve recipients prior to challenge with H1N1pdm or H3N2. Compared with PBS controls, mice receiving immune sera exhibited reduced body weight loss, improved survival, and lower lung viral titers at 3 dpc (Figure 4E–J). These effects were more pronounced with sera from high‐dose vaccines, suggesting that non‐neutralizing antibody functions may contribute to heterologous protection.

**Figure 5:**
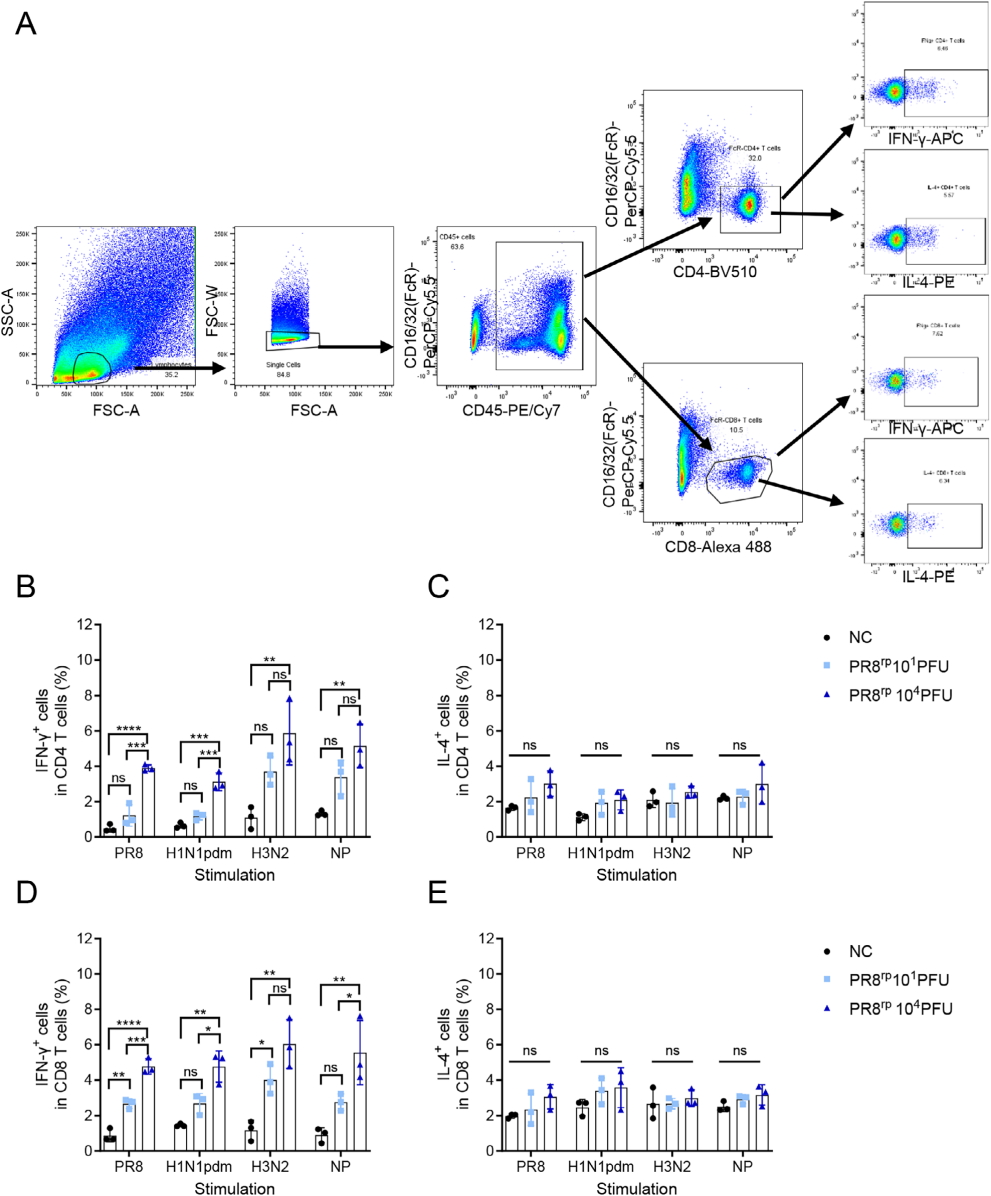
PR8^rp^ elicits functional homologous and heterologous cellular immune responses in mice. BALB/c mice (*n* = 3) were intranasally inoculated with a low (10 PFU) or high (10^4^ PFU) dose of PR8^rp^, or with PBS as a no‐vaccine control (NC). Four weeks later, splenocytes were harvested and stimulated ex vivo with inactivated PR8, A/California/04/2009 (H1N1pdm), A/Aichi/2/1968 (H3N2), or an NP peptide pool. Intracellular cytokine staining was performed to quantify antigen‐specific CD4⁺ and CD8⁺ T cell responses. A) Gating strategy for identifying cytokine‐secreting CD4⁺ and CD8⁺ T cells. B,C) Frequencies of CD4⁺ T cells producing IFN‐γ (B) or IL‐4 (C). D,E) Frequencies of CD8⁺ T cells producing IFN‐γ (D) or IL‐4 (E). Data represent mean ± SEM. *p*‐values were calculated using one‐way analysis of variance (ANOVA) followed by Tukey's multiple‐comparison post hoc test. ns, not significant; *, *p* < 0.05; **, *p* < 0.01; ***, *p* < 0.001; ****, *p* < 0.0001.

We also examined T cell responses by intracellular cytokine staining (ICS) of splenocytes harvested four weeks post‐vaccination. Upon restimulation with homologous inactivated PR8, heterologous H1N1pdm, heterosubtypic H3N2, or an NP peptide pool, PR8^rp^‐vaccinated mice displayed increased frequencies of IFN‐γ–producing CD4⁺ and CD8⁺ T cells compared to controls, with higher responses in the high‐dose group (**Figure** **5**; Figures S2,S3, Supporting Information). IL‐4–producing T cells were not elevated, indicating a Th1‐biased profile.

Together, these humoral and cellular immune readouts serve as immunological correlates of the protection conferred by PR8^rp^, with neutralizing antibodies accounting for homologous protection and a combination of non‐neutralizing antibody activity and cross‐reactive T cell responses likely contributing to heterologous protection.

### 2.5 Defective Genome Packaging, Rather than Impaired RNA or Protein Synthesis, Drives PR8^rp^ Attenuation

**Figure 6:**
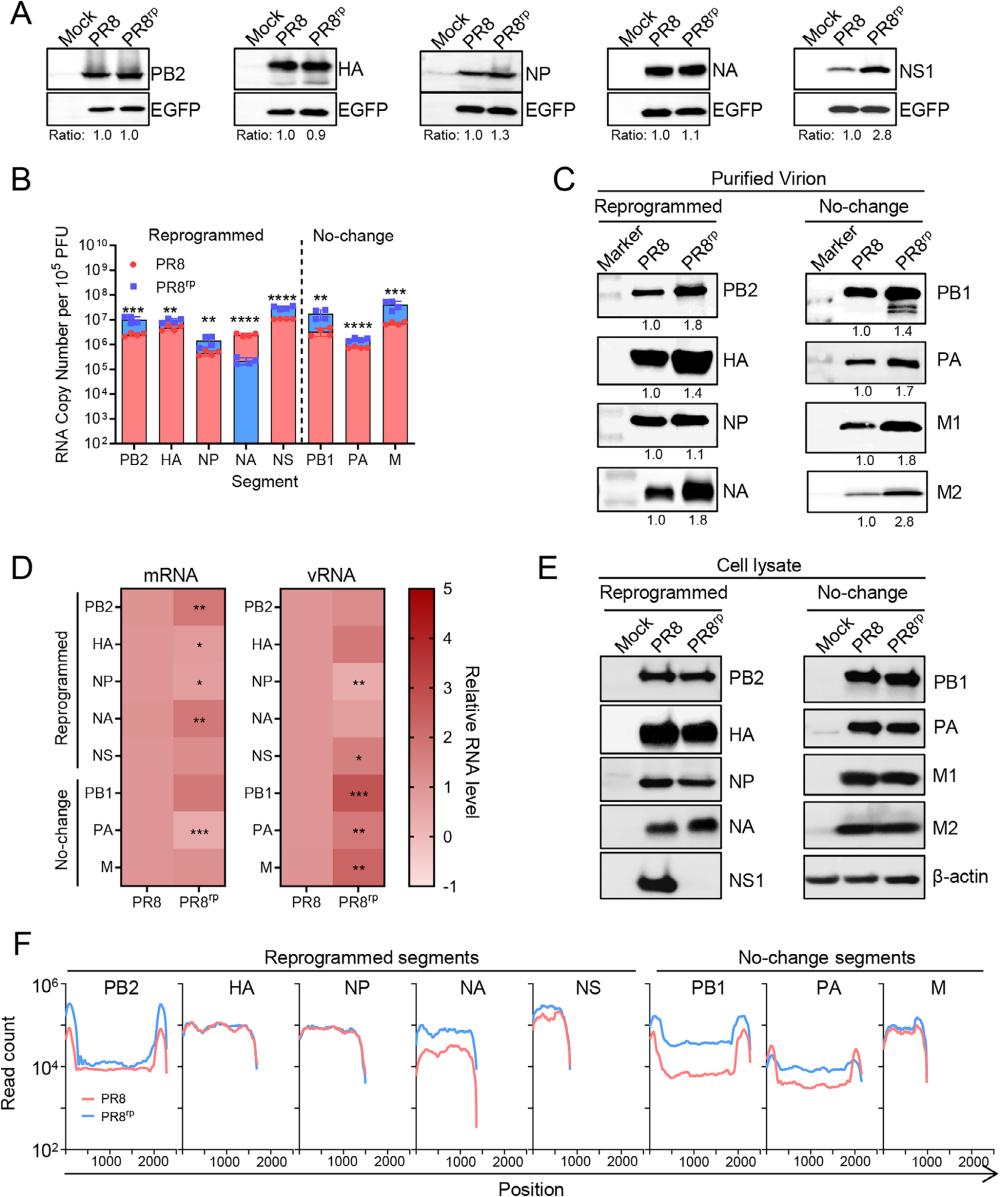
Genome packaging defects, but not RNA or protein synthesis changes, underlie PR8^rp^ attenuation A) Translation efficiency of wild‐type (PR8) and reprogrammed (PR8^rp^) PB2, HA, NP, NA, and NS segments outside infection. HEK293T cells were transfected with bicistronic plasmids expressing each segment together with EGFP; protein expression was analyzed by Western blot at 24 h post‐transfection. Ratios indicate protein levels normalized to EGFP and expressed relative to PR8. B) Segment‐specific vRNA copy numbers in purified virions (10^5^ PFU), determined by qRT‐PCR. Data are presented as mean ± SD. C) Western blot analysis of structural proteins in purified virions; ratios indicate levels relative to PR8. D) Segment‐specific mRNA and vRNA levels in MDCK cells infected at MOI = 3 for 6 h, quantified by qRT‐PCR and expressed relative to PR8. E) Viral protein expression in infected cell lysates (MOI = 3) at 6 hpi; β‐actin served as loading control. F) Genome coverage profiles from high‐throughput sequencing of infected MDCK cells at MOI = 3 for 6 h. Data are shown as mean read counts from three independent biological replicates. *p*‐values were calculated using unpaired two‐tailed Student's *t‐*tests. *, *p *< 0.05; **, *p *< 0.01; ***, *p *< 0.001; ****, *p *< 0.0001.

To dissect the molecular basis of PR8^rp^ attenuation from the perspective of the virus itself, we first assessed whether codon reprogramming altered the translation efficiency of the five reprogrammed gene segments (PB2, HA, NP, NA, and NS) outside the context of infection. Each wild‐type or reprogrammed ORF was cloned into a bicistronic plasmid co‐expressing EGFP as a transfection control. Plasmids were transfected into HEK293T cells, and protein expression was examined 24 h post‐transfection by Western blot. Overall, most reprogrammed proteins were expressed at levels similar to their wild‐type counterparts, except NS1, which showed a ≈2.8‐fold increase in expression (**Figure** **6**A). These results indicate that codon reprogramming generally did not impair basal translation efficiency, though the NS1 segment displayed an unexpected increase.

We next investigated whether codon reprogramming affected viral genome packaging. Using quantitative RT‐PCR on purified virions with equal infectious titer of 10^5 ^PFU, we found that PR8^rp^ virions contained significantly higher vRNA levels for PB2, PB1, PA, HA, NP, M, and NS segments compared to wild‐type PR8, with increases ranging from 1.6‐ to 5.3‐fold (*p* = 0.0079 to *p* < 0.0001) (Figure 6B). In contrast, the reprogrammed NA segment exhibited an 11.5‐fold decrease in vRNA packaging efficiency (*p *< 0.0001), consistent with our deliberate design to retain only the minimal packaging signal in NA, as in our previous work, to impair its incorporation.^[^ ^16^ ^]^ This defect likely increases the proportion of non‐infectious particles in the PR8^rp^ population, thereby reducing overall viral fitness. Western blot analysis of purified virions further supported this conclusion, revealing elevated levels of all structural proteins relative to PR8 (Figure 6C), indicative of an increased non‐infectious to infectious particle ratio.

We then examined whether codon reprogramming affected viral RNA and protein synthesis during a single replication cycle. MDCK cells were infected with PR8 or PR8^rp^ at an MOI of 3, and viral RNA and protein levels were measured at 6 hpi. Quantification of segment‐specific mRNA and vRNA, together with Western blot analysis of the corresponding proteins, showed only small differences between PR8^rp^ and PR8 for most gene segments (Figure 6D,E); although some changes reached statistical significance, their magnitudes were minimal, indicating that transcription, genome replication, and overall protein expression remained largely intact (Figure 6D,E). High‐throughput sequencing further confirmed uniform full‐length coverage across all segments, with PR8^rp^ displaying similar or slightly higher read counts for the reprogrammed genes compared with PR8 (Figure 6F), suggesting that synonymous recoding did not impair complete genome synthesis. A notable exception was NS1, whose protein expression in PR8^rp^‐infected cells was nearly undetectable despite comparable mRNA levels, suggesting a post‐transcriptional defect. This impaired NS1 production may contribute to PR8^rp^ attenuation, given NS1's well‐established role in counteracting host antiviral responses.^[^ ^26^ ^]^

### 2.6 Codon Reprogramming Enhances Host Antiviral Responses and Increases PR8^rp^ Susceptibility to ZAP‐Mediated Restriction

**Figure 7:**
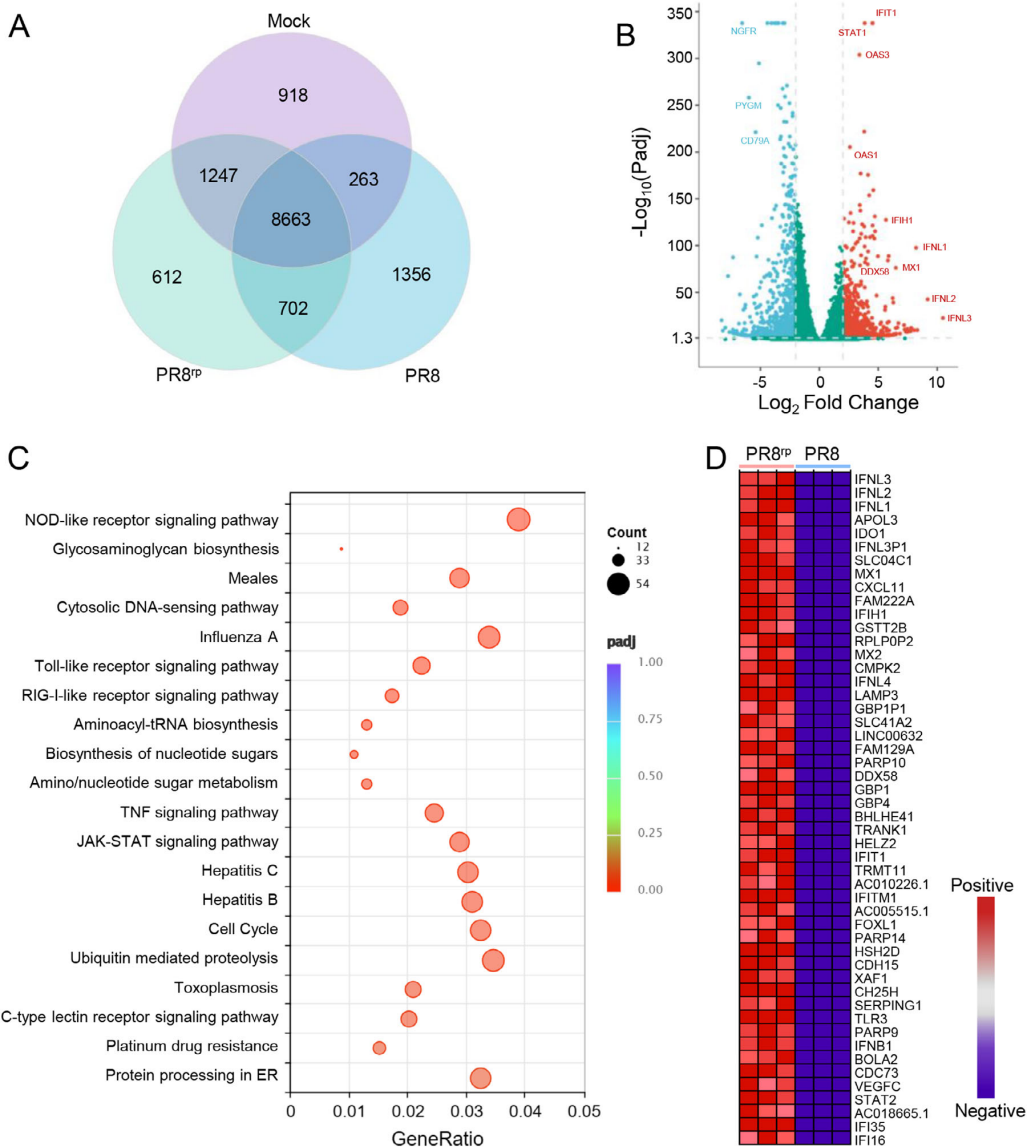
Transcriptomic profiling reveals enhanced activation of host antiviral responses by PR8^rp^. A549 cells were infected with PR8 or PR8^rp^ at an MOI of 3 for 12 h, with mock‐infected cells serving as controls. A) Venn diagram showing the overlap of expressed genes among mock‐, PR8‐, and PR8^rp^‐infected cells. B) Volcano plot of differentially expressed genes between PR8^rp^‐ and PR8‐infected cells (padj < 0.05, |log_2_ fold change| ≥ 2). Red and blue dots represent significantly upregulated and downregulated genes, respectively; green dots indicate genes with no significant change. C) Bubble plot of KEGG pathway enrichment analysis of genes upregulated in PR8^rp^ relative to PR8 infection. Dot color represents adjusted *p*‐value, and dot size indicates gene count per pathway. D) Heatmap of the top 50 features for the PR8^rp^ group in differentially expressed genes based on Gene Set Enrichment Analysis.

Given the marked reduction in NS1 protein expression, we next examined whether PR8^rp^ infection triggered stronger host antiviral responses. Transcriptomic profiling was performed on A549 cells infected with PR8 or PR8^rp^ (MOI = 3) for 12 h. The analysis revealed that although most transcripts were shared between the two infections, PR8^rp^ induced a larger set of unique genes and showed a greater number of significantly upregulated genes compared with PR8 (**Figure** **7**A,B). Many of these upregulated genes were antiviral effectors. KEGG pathway enrichment analysis highlighted significant activation of innate immune–related pathways, including JAK–STAT signaling, RIG‐I–like receptor signaling, Toll‐like receptor signaling, and cytosolic DNA‐sensing pathways (Figure 7C). Further differential pathway analysis identified the top antiviral gene sets preferentially enriched in PR8^rp^‐infected cells (Figure 7D). Heatmap visualization of the top 50 upregulated genes underscored their distinct expression profiles compared with PR8 infection. Strikingly, PR8^rp^ infection led to a higher induction of multiple type I and III interferons (e.g., IFN‐β, IFN‐λ1, IFN‐λ 2, IFN‐λ3), accompanied by broad upregulation of interferon‐stimulated genes (ISGs), including MX1, ISG15, the OAS family (OAS1, OAS2, OASL), and IFIT family members (IFIT1, IFIT2, IFIT3). Collectively, these findings indicate that PR8^rp^ infection more strongly activates host antiviral transcriptional programs compared with PR8.

**Figure 8:**
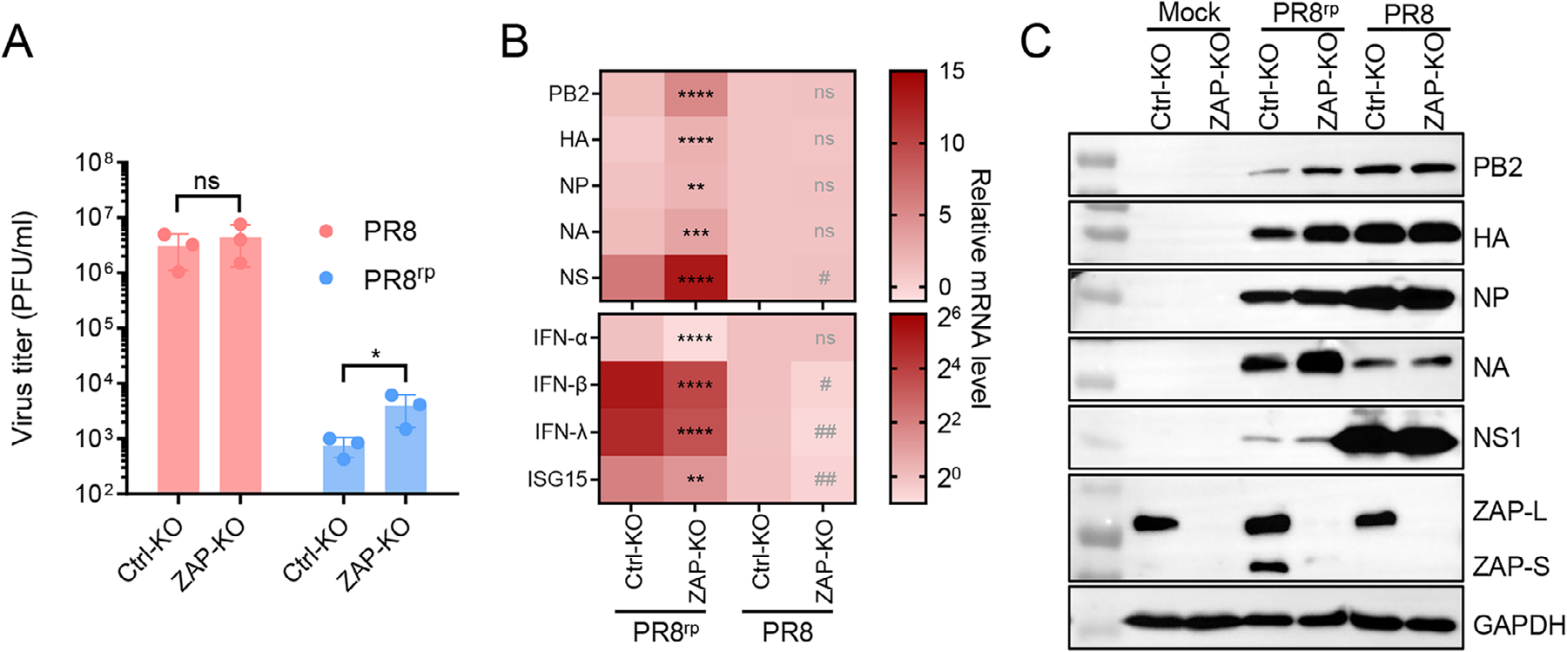
ZAP restricts PR8^rp^ replication by targeting CpG‐enriched viral RNA and enhancing antiviral gene expression. A) Replication of PR8 and PR8^rp^ in CRISPR‐Cas9–generated ZAP‐knockout (ZAP‐KO) or control knockout (Ctrl‐KO) A549 cells. Cells were infected at an MOI of 0.01, and supernatant virus titers at 24 hpi were quantified by plaque assay. Data are shown as mean ± SEM. B) Heatmap of relative mRNA levels for viral genes (top) and antiviral host genes (bottom) in PR8^rp^‐ or PR8‐infected ZAP‐KO and Ctrl‐KO cells, normalized to GAPDH and expressed relative to PR8‐infected Ctrl‐KO cells. Values represent means of three biological replicates. *p*‐values were calculated using unpaired two‐tailed Student's *t*‐tests. Asterisks indicate statistically significant differences in the indicated mRNA levels between PR8^rp^‐infected ZAP‐KO cells and PR8^rp^‐infected Ctrl‐KO cells; pound symbols indicate statistically significant differences in the indicated mRNA levels between PR8‐infected ZAP‐KO cells and PR8‐infected Ctrl‐KO cells. ns, not significant; * or #, *p* < 0.05; ** or ##, *p* < 0.01; ***, *p* < 0.001; ****, *p* < 0.0001. C) Western blot analysis of viral proteins (PB2, HA, NP, NA, NS1) and ZAP isoforms (ZAP‐L, ZAP‐S) in lysates from PR8^rp^‐ or PR8‐infected ZAP‐KO and Ctrl‐KO cells at 24 hpi. GAPDH served as a loading control.

ZAP selectively recognizes and degrades viral RNAs enriched in CpG dinucleotides.^[^ ^20^, ^27^, ^28^ ^]^ As codon reprogramming in PR8^rp^ increased CpG content (Table 1), we hypothesized that ZAP contributes to its attenuation. Using CRISPR‐Cas9–generated ZAP‐knockout (ZAP‐KO) A549 cells, we found that PR8^rp^ replication was significantly enhanced in the absence of ZAP (*p* = 0.0349), whereas wild‐type PR8 was unaffected (**Figure** **8**A), indicating the sensitivity of PR8^rp^ to ZAP‐mediated restriction. In PR8^rp^‐infected cells, ZAP knockout markedly elevated mRNA levels (6.6–66.4‐fold, *p* = 0.0044–*p* < 0.0001) and protein production for reprogrammed segments PB2, HA, NP, and NA, but not NS1, whose protein expression defect persisted (Figure 8B,C). For PR8, viral gene expression was largely unchanged by ZAP status. Consistent with ZAP's role in amplifying RIG‐I–mediated type I IFN responses,^[^ ^24^ ^]^ ZAP knockout reduced IFN‐α, IFN‐β, IFN‐λ, and ISG15 expression, with a greater decrease in PR8^rp^‐infected cells (Figure 8B, bottom panel), suggesting a stronger ZAP dependency for IFN induction. Together, these data indicate that ZAP restricts PR8^rp^ replication by targeting CpG‐enriched viral RNA and enhancing antiviral gene expression.

### 2.7 Applicability of the Codon Reprogramming Strategy to a Contemporary H1N1 Strain

To test whether our codon reprogramming approach is transferable beyond the prototype PR8 strain, we applied it to a recent circulating H1N1 isolate, A/Victoria/4897/2022 (Vic4897). Following the same design principles as PR8^rp^, we introduced 449 and 393 synonymous mutations into the HA and NA segments, respectively (Table S3, Supporting Information), markedly increasing CpG content and reducing the codon adaptation index (CAI) relative to influenza virus codon usage, while maintaining a comparable CAI to human codon usage.

The reprogrammed Vic4897 HA and NA segments were combined with the reprogrammed PB2, NP, and NS segments from PR8^rp^, along with wild‐type PB1, PA, and M segments from PR8, to generate Vic4897^rp^ (Figure S1A, Supporting Information). Vic4897^rp^ exhibited clear attenuation, producing smaller plaques than wild‐type Vic4897 (Figure S1B, Supporting Information) and showing 2–3 log_10_ lower titers in embryonated eggs, MDCK cells, and A549 cells across the infection time course (Figure S1C–E, Supporting Information). These results demonstrate that our codon reprogramming strategy can be readily applied to contemporary circulating influenza viruses to achieve robust attenuation.

## 3 Discussion

We designed PR8^rp^ by replacing five gene segments with sequences extensively recoded to use the influenza virus's least‐preferred synonymous codons, thereby introducing ≈2000 silent mutations and markedly increasing CpG content. This represents the highest synonymous mutation load yet reported for influenza and produced profound attenuation in vitro and in vivo without compromising immunogenicity. Distributing this extreme mutation load across multiple essential segments greatly reduces the probability of reversion to virulence, addressing a major safety concern for live attenuated vaccines. By imposing multiple independent fitness constraints through intrinsic viral defects and heightened innate immune restriction, PR8^rp^ maintained broad protective efficacy against homologous, heterologous, and heterosubtypic challenge while achieving a vastly improved safety margin over the parental PR8 strain.

Our work extends earlier synonymous recoding strategies that targeted single segments (e.g., NS with 113 mutations^[^ ^11^ ^]^) or used genome‐scale recoding yet with fewer substitutions, such as avian influenza‐preferred codons (373 mutations^[^ ^13^ ^]^) or codon‐pair deoptimization limited to certain segments (up to 903 mutations^[^ ^10^, ^17^, ^19^, ^20^ ^]^). By deliberately using codons most disfavored by influenza, we achieved a substantially higher permissible mutation load without altering amino acid sequences or disrupting essential packaging signals. Distributing these changes across multiple segments likely imposes several independent fitness constraints, contributing to the pronounced attenuation and enhanced safety profile observed.

Mechanistic analysis revealed that attenuation was multifactorial. First, deliberate modification of the NA packaging signal reduced NA vRNA incorporation into virions by greater than tenfold, likely increasing the ratio of non‐infectious to infectious particles and diminishing transmission competence, which is in line with our previous work.^[^ ^16^ ^]^ Second, PR8^rp^ exhibited a segment‐specific post‐transcriptional defect in NS1 protein accumulation, despite unaltered NS mRNA levels, a phenotype not rescued by ZAP knockout. Given NS1's role in suppressing type I/III interferon signaling,^[^ ^26^, ^29^, ^30^ ^]^ this defect likely sensitizes PR8^rp^ to innate immune responses. Third, codon reprogramming markedly increased CpG content, rendering PR8^rp^ sensitive to ZAP‐mediated restriction. In ZAP‐deficient cells, PR8^rp^ replication and protein expression were substantially restored, and interferon induction was reduced, indicating that ZAP acts both as a direct antiviral effector and an amplifier of antiviral signaling. These combined effects — impaired NA packaging, loss of NS1‐mediated immune evasion, and CpG‐triggered ZAP restriction — together explain the sharp dorp in replication fitness.

Interestingly, codon reprogramming did not globally suppress transcription or translation of other segments under transfection and single‐cycle conditions. The largely uncompromised protein expression of most reprogrammed genes is consistent with their calculated CAIs relative to the human host, which were maintained at moderate or high levels despite extensive recoding. This indicates that attenuation is not driven by slowed protein synthesis, as has been proposed for some codon‐deoptimized viruses,^[^ ^19^ ^]^ but rather by targeted functional defects and enhanced innate immune recognition. An additional advantage of this strategy is that it preserves sufficient antigen expression during vaccination, ensuring adequate immunogen availability and effective antigen delivery to elicit protective immune responses.

Importantly, the preservation of robust antigen expression in PR8^rp^ translated into strong protective immunity in vivo. Despite its attenuation, PR8^rp^ induced sterilizing immunity against homologous PR8 challenge, even at doses as low as 1 PFU. This efficacy was associated with strong HI and MN antibody responses. Notably, PR8^rp^ also conferred substantial cross‐protection against H1N1pdm and H3N2 viruses, which lack antigenic match to PR8. Passive serum transfer experiments indicate that this heterologous protection may involve non‐neutralizing antibody functions, while T cell profiling revealed robust IFN‐γ–producing CD4⁺ and CD8⁺ responses with a Th1 bias, consistent with cross‐reactive cellular immunity targeting conserved internal antigens. These findings align with prior evidence that live‐attenuated influenza vaccines can elicit broader immunity than inactivated vaccines.^[^ ^31^, ^32^ ^]^

One limitation of PR8^rp^ is its reduced yield in eggs and MDCK cells compared with PR8. However, the low dose needed for effective intranasal immunization (<10 PFU) greatly reduces per‐dose production requirements. In practice, egg‐based manufacturing could still supply adequate doses for large‐scale immunization. Furthermore, our proof‐of‐concept with Vic4897^rp^ demonstrates that this recoding platform is adaptable to contemporary circulating strains, supporting its potential as a flexible vaccine design strategy.

Beyond the scope of influenza, this genome‐wide codon reprogramming strategy offers broad utility as a rapid‐response platform for live attenuated vaccine development and virological research.^[^ ^18^, ^33^ ^]^ Because it relies solely on synonymous substitutions without altering amino acid sequences, it can be readily adapted to diverse RNA viruses, enabling the swift generation of vaccine candidates against emerging and re‐emerging pathogens such as SARS‐CoV‐2,^[^ ^15^, ^34^ ^]^ pandemic‐potential avian influenza, and other zoonotic threats. The ability to impose multiple independent fitness constraints while preserving antigen expression is particularly advantageous for emergency preparedness, where both attenuation and immunogenicity are essential. Furthermore, reprogrammed viruses generated using this approach can serve as precise tools for dissecting virus–host interactions, replication dynamics, and immune evasion mechanisms, thereby advancing both applied vaccine platforms and fundamental understanding of viral biology.^[^ ^33^ ^]^

In conclusion, PR8^rp^ represents a next‐generation live attenuated influenza vaccine candidate with unprecedented synonymous mutation load, multifactorial attenuation, and broad protective potential. Its design principles are broadly applicable, offering a rational framework for safe, effective, and adaptable LAIVs against influenza.

## 4 Experimental Section

### Ethics Statement

Animal experiments were approved by the Institutional Animal Care and Use Committee of Guangzhou Medical University (Permit No. 202 014 and 202 040) and performed in an Animal Biosafety Level 2 (ABSL‐2) facility at the First Affiliated Hospital of Guangzhou Medical University.

### Cells and Viruses

Madin‐Darby Canine Kidney (MDCK) cells (CCL‐34, American Type Culture Collection [ATCC]), human embryonic kidney (HEK) 293H cells (Invitrogen, Thermo Fisher Scientific), HEK 293T cells (CRL‐3216, ATCC), and A549 human lung carcinoma cells were cultured in Dulbecco's Modified Eagle medium (DMEM, Gibco) supplemented with 10% fetal bovine serum (FBS, Gibco) at 37 °C with 5% CO_2_.

The A/PR/8/34 (H1N1) (PR8) (VR‐95PQ, ATCC) and A/Aichi/2/68 (H3N2) (VR‐547, ATCC) were maintained in the laboratory. The A/California/04/2009 (H1N1pdm) strain was kindly provided by Dr. Yi Shi (Chinese Academy of Sciences). All viral stocks were verified by Sanger sequencing and propagated in 10‐day‐old embryonated chicken eggs.

ZAP‐knockout (ZAP‐KO) cells were generated as previously described.^[^ ^35^ ^]^ Briefly, single guide RNAs (sgRNAs) targeting ZAP (5′‐TTTGTGGTGTTGGAGACCGG‐3′) and GFP (5′‐GAAGTTCGAGGGCGACACCC‐3′; used as a non‐targeting control) were designed using the CRISPR guide design tool (https://zlab.bio/guide‐design‐resources). sgRNA sequences were cloned into the LentiCRISPR V2 vector (Addgene, Cat. No. 52 961) via *BsmBI* restriction sites. Lentiviruses were packaged in HEK293T cells and used to transduce wild‐type A549 cells. Polyclonal ZAP‐KO and GFP‐KO (Ctrl‐KO) A549 cells were generated via lentiviral transduction and subsequent puromycin selection, and ZAP knockout was validated by western blotting.

### Viral Segment Reprogramming and Virus Rescue

The coding regions of each gene segment from the PR8 strain were reprogrammed to rearrange synonymous codons according to their relative rarity in influenza A virus genomes, as defined by the influenza codon‐usage frequency matrix reported in ref. [36] For each amino acid, codons with the lowest usage frequency in influenza viruses (e.g., GCG for alanine, GGC for glycine) were selected to replace the original codons, while maintaining the original amino acid sequence. The substitution rules for all amino acids are listed in Table S1 (Supporting Information). Codon substitutions were designed using an in‐house codon reprogramming code (available on GitHub at https://github.com/Wang‐Yang‐hub/CodonReprogramming.git), which automatically replaced codons according to these predefined rules. Packaging signals located at the 5′ and 3′ ends of each coding region were excluded from codon substitutions to preserve genome packaging efficiency (specific substitution regions are indicated in Figure 1A). Reprogrammed gene segments were synthesized de novo (Synbio, Suzhou, China) and cloned into a standard ambisense eight‐plasmid reverse genetics system, as described previously.^[^ ^37^, ^38^ ^]^ Recombinant PR8^rp^ viruses carrying reprogrammed PB2, HA, NP, NA, and NS segments, along with wild‐type PB1, PA, and M segments from PR8, were rescued by reverse genetics. For the Vic4897^rp^ generation, the HA and NA segments of A/Victoria/4897/2022 (H1N1) were reprogrammed using the same codon replacement rules as PR8^rp^. The virus was rescued by combining these reprogrammed HA and NA segments with reprogrammed PB2, NP, and NS from PR8^rp^, and wild‐type PB1, PA, and M from PR8.

### Viral Growth Kinetics

To assess viral growth kinetics in cell culture, MDCK or A549 cells were infected with the indicated viruses at a multiplicity of infection (MOI) of 0.01. After 1 h incubation at 37 °C, cells were washed with PBS and maintained in viral growth medium (DMEM supplemented with 0.3% bovine serum albumin [BSA; MP Biomedicals], 1 µg mL^−^ TPCK‐treated trypsin [Sigma‐Aldrich], and 1% penicillin–streptomycin [Invitrogen]) at 37 °C with 5% CO_2_. Supernatants were collected at the indicated time points, and virus titers were determined by immunostaining‐based plaque assays on MDCK cells, as previously described.^[^ ^16^ ^]^ To determine viral growth in embryonated eggs, 10‐day‐old specific pathogen‐free embryonated chicken eggs were inoculated with 100 PFU of each virus. Eggs were incubated at 37 °C, and allantoic fluids were harvested at the indicated time points post‐infection. Viral titers in the allantoic fluid were measured by plaque assay on MDCK cells. The detection limit was 10 PFU mL^−1^; undetectable samples were assigned a value of 5 for statistical analyses.

### Animal Studies

To evaluate viral attenuation, six‐week‐old female BALB/c mice were anesthetized with isoflurane and intranasally infected with indicated doses of virus in a 50 µL volume. As a control, a group of mice received an equivalent volume of PBS. Body weight and survival were monitored daily for 14 days. Mice exhibiting severe illness or ≥25% body weight loss were euthanized. The 50% lethal dose (LD_50_) values were calculated using the Reed–Muench method. For assessing viral replication in the respiratory tract of mice, lung tissues were collected 3 days post‐infection. Lungs were homogenized in DMEM containing 0.3% BSA, and 1% penicillin–streptomycin, and virus titers were determined by plaque assay and TCID_50_ assay.

To assess vaccine effectiveness, six‐week‐old female BALB/c mice were intranasally immunized with the indicated doses of virus in 50 µL PBS. Four weeks later, sera and spleens were collected from five mice per group for antibody and cellular immune response assays. Thirty days post‐vaccination, mice were challenged intranasally with 100 × LD_50_ of PR8, 10 × LD_50_ of mouse‐adapted A/California/04/2009 (H1N1pdm), or 3 × LD_50_ of A/Aichi/2/68 (H3N2). Survival and body weight were monitored for 14 days post‐challenge. Lungs were collected at 3 and 5 days post‐challenge for virus titration and histopathology.

### Histopathology

Lungs were fixed in 10% PBS‐buffered formalin, embedded in paraffin, and sectioned at 5 µm thickness. Sections were stained with hematoxylin and eosin (H&E) and examined under a light microscope (Nikon).

### Hemagglutination Inhibition Assay

Hemagglutination inhibition (HI) assay was performed as previously described.^[^ ^38^ ^]^ In brief, sera were treated with receptor‐destroying enzyme II (RDE II) and two‐fold serially diluted in V‐bottom plates. Four hemagglutination units (HAU) of the designated virus were added to each well and incubated for 30 min at room temperature. Then, 1% chicken red blood cells were added and incubated for an additional 30 min. HI titers were recorded as the reciprocal of the highest serum dilution preventing hemagglutination. The detection limit was 20; undetectable samples were assigned a value of 10 for statistical analyses.

### Micro‐Neutralization Assay

Micro‐neutralization (MN) assays were performed as described previously,^[^ ^38^ ^]^ with slight modifications. RDE II‐treated sera were twofold serially diluted in flat‐bottom 96‐well plates and mixed with 100 TCID_50_ of the indicated virus. After 1 h incubation at 37 °C, MDCK cells (3 × 10^4^ well^−1^) were added and incubated for 24 h. Cells were fixed with 80% acetone, and viral NP was detected by ELISA using a polyclonal anti‐influenza A NP antibody (Cat. No. 11675‐T62‐100, Sinobio). MN titers were defined as the reciprocal of the highest serum dilution that neutralized ≥ 50% of viral infection. The detection limit was 20; undetectable samples were assigned a value of 10 for statistical analyses.

### Serum Passive Transfer Experiment

Sera collected from immunized mice were subjected to a 30‐minute heat treatment at 56 °C to deactivate the complement system. These treated sera were then administered intraperitoneally (i.p.) to naive BALB/c mice. Two hours later, mice were challenged with 10 × LD_50_ of the mouse‐adapted A/California/04/2009 (H1N1pdm), or 3 × LD_50_ of A/Aichi/2/1968 (H3N2) viruses. Survival and body weight were monitored for 14 days. Lungs were collected at 3 days post‐challenge for virus titration.

### Flow Cytometry and Intracellular Cytokine Staining (ICS)

Flow cytometry and ICS were performed as described previously^[^ ^39^ ^]^ with minor modifications. Spleens from immunized mice were mechanically dissociated in cold PBS and filtered through 70‐µm cell‐strainers. Red blood cells were lysed with ACK buffer, and splenocytes were resuspended in RPMI 1640 medium (Gibco). For antigen‐specific stimulation, splenocytes were stimulated for 5–6 h at 37 °C in 5% CO_2_ with either 2 µg mL^−1^ NP peptide pool or purified inactivated viruses (10 µg mL^−1^ total protein), in the presence of brefeldin A (BD Biosciences) to block cytokine secretion. Following stimulation, cells were blocked with anti‐mouse CD16/32‐PerCP‐Cy5.5 (clone 93, Cat. No. 45‐0161‐82, eBioscience) for 15 min at 4 °C to prevent nonspecific Fc binding. Surface staining was then performed using anti‐mouse CD45‐PE/Cy7 (clone 30‐F11, Cat. No. 103 113, Biolegend), anti‐mouse CD4‐eFluor 450 (clone RM4‐5, Cat. No. 48‐0042‐82, eBioscience), and anti‐mouse CD8a‐Alexa 488 (clone 53–6.7, Cat. No. 100 723, Biolegend) antibodies. Cells were then fixed and permeabilized using the Cytofix/Cytoperm Kit (Cat. No. 554 714, BD Biosciences) according to the manufacturer's protocol. Intracellular cytokines were stained with anti‐mouse IFN‐γ‐APC (clone XMG1.2, Cat. No. 17‐7311‐82, eBioscience) and anti‐mouse IL‐4‐PE (clone 11B11, Cat. No. 504 103, Biolegend). Data acquisition was performed on a BD FACSVerse flow cytometer (BD Biosciences), and analysis was conducted using FlowJo 10 software (Tree Star). Cytokine‐positive CD4⁺ and CD8⁺ T cell populations were identified after gating on live, singlet, CD45⁺ lymphocytes.

### Transient Transfection

The coding regions of viral genes (PB2, HA, NP, NA, NS1; wild‐type or reprogrammed) were individually cloned into the bicistronic reporter plasmid pVITRO2 (Cat. No. pvitro2‐nmcsm, InvivoGen). In each construct, the viral gene was placed under the control of the hFerL promoter, and the EGFP gene under the control of the FerH promoter. The obtained dual‐expression plasmids were transfected into HEK293T cells using Lipofectamine 2000 (Thermo Fisher Scientific), and protein expression was assessed by Western blot 24 h post‐transfection.

### Western Blot

To assess protein expression following transfection or infection, cells were lysed in RIPA buffer (Cat. No. P0013, Beyotime) containing PMSF, mixed with SDS loading buffer containing 1% β‐mercaptoethanol, and boiled for 10 min. For purified virions, samples were prepared similarly in SDS loading buffer with 1% β‐mercaptoethanol and boiled for 10 min. Proteins from cell lysates or virions were separated by SDS–PAGE under reducing conditions and transferred to PVDF membranes (Cat. No. IPVH00010, Millipore). Membranes were blocked with 5% non‐fat milk in PBS containing 0.05% Tween‐20 for 1 h at room temperature, then incubated overnight at 4 °C with primary antibodies against PB2 (Cat. No. GTX125926, GeneTex), PB1 (Cat. No. GTX125923, GeneTex), PA (Cat. No. GTX125932, GeneTex), HA (Cat. No. GTX127357, GeneTex), NP (Cat. No. 11675‐T62‐100, Sinobio), NA (Cat. No. GTX125974, GeneTex), M1 (Cat. No. NBP2‐14995, Novus), M2 (Cat. No. NBP2‐14997, Novus), NS1 (Cat. No. M100035, Zoonogen), EGFP (Cat. No. R24437, Zenbio), GAPDH (Cat. No.60004‐1‐Ig, Proteintech) or β‐actin (Cat. No. R380624, Zenbio). After washing, membranes were incubated with HRP‐conjugated species‐specific secondary antibodies (Cat. Nos. E030110‐02 and E030120‐02, EARTH), and signals were detected using an ECL reagent (Cat. No. G2020‐500ML, Servicebio).

### RNA Extraction and Quantitative RT‐PCR

Total RNA from cells and viral RNA from purified virions were extracted using the VAMNE Magnetic Cell/Tissue Total RNA Kit (Cat. No. RMA101‐C2‐P3, Vazyme) and VAMNE Virus DNA/RNA Extraction Kit (Cat. No. RM501‐02, Vazyme), respectively. Quantification of viral vRNA and mRNA expression levels in infected cells was performed as previously described.^[^ ^40^ ^]^ Briefly, cDNA synthesis for vRNA and mRNA was performed using tagged primers (Table S4, Supporting Information). Quantitative PCR (qPCR) was performed using ChamQ Universal SYBR qPCR Master Mix (Cat. No. Q711‐02, Vazyme) on an Applied Biosystems QuantStudio 5 system. For the analysis of host gene expression, cDNA was synthesized using oligo(dT) primers. To quantify vRNA levels in purified virions, cDNA synthesis was carried out using the universal Uni12 primer (5′‐AGCAAAAGCAGG‐3′). All primer sequences used in this study are listed in Table S4 (Supporting Information). Relative gene expression levels were calculated using the 2^−ΔΔCT^ method. The copy numbers were quantified using standard curves generated from known concentrations of pVITRO2 constructs containing individual viral genes.

### RNA Sequencing

A549 or MDCK cells were infected with the indicated viruses at an MOI of 3 and harvested at 12 and 6 hpi, respectively. Total RNA was extracted using the VAMNE Magnetic Cell/Tissue Total RNA Kit (Cat. No. RMA101‐C2‐P3, Vazyme). mRNA‐sequencing libraries were prepared using the NEBNext Ultra RNA Library Prep Kit for Illumina (NEB), following the manufacturer's instructions. Sequencing was performed on the NovaSeq 6000 platform (Illumina, USA) with 150 bp paired‐end reads. The raw reads were subjected to quality filtering resulting in high‐quality clean reads for downstream analyses. The clean reads were aligned to the reference genome using Hisat2 v2.0.5. Differential expression analysis was performed using the DESeq2 R package v1.20.0. genes with an absolute log_2_ fold change (|log_2_FC|) ≥ 1 and an adjusted *p*‐value (padj) ≤ 0.05 were assigned as differentially expressed. Significant differentially expressed genes (DEGs) were subjected to Kyoto Encyclopedia of Genes and Genomes (KEGG) pathway enrichment analysis using the ClusterProfiler v3.8.1. Additionally, Gene Set Enrichment Analysis (GSEA) was performed with gsea v3.0 on the entire ranked gene list based on log_2_ fold change. Viral RNA reads were assembled via reference‐guided genome reconstruction using IRMA (Iterative Refinement Meta‐Assembler) version 1.1.5.^[^ ^41^ ^]^

### Statistical Analysis

Statistical analyses were performed using GraphPad Prism 10 (GraphPad Software). For comparisons between two groups with equal variances, unpaired two‐tailed Student's *t*‐tests were used. For comparisons among more than two groups with equal variances, one‐way analysis of variance (ANOVA) followed by Tukey's multiple‐comparison post hoc test was applied. For analysis of growth curves, two‐way ANOVA with Šidák's multiple‐comparison correction was used to compare group means at each time point. For analysis of body weight changes over time, two‐way repeated‐measures ANOVA with Tukey's multiple‐comparison post hoc test was performed to evaluate the main effects among groups. Survival curves were analyzed using the log‐rank (Mantel–Cox) test. Viral titers, RNA copy numbers, and antibody titers were log‐transformed before statistical analysis to meet the assumptions of normality. A *p*‐value < 0.05 was considered statistically significant.

## Conflict of Interest

The authors declare no conflict of interest.

## Supporting information



Supporting Information

## Data Availability

All next‐generation sequencing data generated in this study have been deposited in the NCBI Sequence Read Archive (SRA) under BioProject accession number PRJNA1308186. The data will be publicly released upon acceptance of the manuscript.
