# Supplementary material for: Genome‐Wide Codon Reprogramming Enables a Multifactorially Attenuated Influenza Vaccine with Broad Cross‐Protection
Source: Adv Sci (Weinh). 2025 Nov 30;13(9):e16448. doi: 10.1002/advs.202516448 (PMC12904059; doi:10.1002/advs.202516448)

Supporting Information

**Genome-wide codon reprogramming enables a multifactorially attenuated influenza vaccine with broad cross-protection**

*Yang Wang^*^, Tianxin Ma, Yujiao He, Qinming Li, Kailin Mai, Minying Mo, Chenyang Cao, Jiahui Li, Pei Feng, Jiaojiao Peng, Jing Sun, Weiqi Pan^*^, Zifeng Yang^*^, Ling Chen^*^*

The supporting information includes:

Tables S1 to S4,

Figure S1 to S3.

**Table S1. Codon replacement rules for the viral genome reprogramming of PR8^rp^**

| Amino acid | Original codon | Relative synonymous codon usage frequency^a)^ | | Codon used after reprogramming |
| --- | --- | --- | --- | --- |
|  |  | Homo sapiens | Influenza A virus |  |
| Alanine (Ala) | GCA | 0.23 | 0.43 | GCG |
|  | GCC | 0.4 | 0.22 | GCG |
|  | GCG | 0.11 | 0.08 | No change |
|  | GCT | 0.27 | 0.27 | GCG |
| Cysteine (Cys) | TGC | 0.54 | 0.6 | TGT |
|  | TGT | 0.46 | 0.4 | No change |
| Aspartic Acid (Asp) | GAC | 0.54 | 0.44 | No change |
|  | GAT | 0.46 | 0.56 | GAC |
| Glutamic Acid (Glu) | GAA | 0.42 | 0.56 | GAG |
|  | GAG | 0.58 | 0.44 | No change |
| Phenylalanine (Phe) | TTC | 0.54 | 0.53 | TTT |
|  | TTT | 0.46 | 0.47 | No change |
| Glycine (Gly) | GGA | 0.25 | 0.45 | GGC |
|  | GGC | 0.34 | 0.13 | No change |
|  | GGG | 0.25 | 0.27 | GGC |
|  | GGT | 0.16 | 0.15 | GGC |
| Asparagine (Asn) | AAC | 0.53 | 0.43 | No change |
|  | AAT | 0.47 | 0.57 | AAC |
| Proline (Pro) | CCA | 0.28 | 0.39 | CCG |
|  | CCC | 0.32 | 0.19 | CCG |
|  | CCG | 0.11 | 0.12 | No change |
|  | CCT | 0.29 | 0.3 | CCG |
| Glutamine (Gln) | CAA | 0.26 | 0.55 | CAG |
|  | CAG | 0.74 | 0.45 | No change |
| Arginine (Arg) | AGA | 0.22 | 0.47 | CGT |
|  | AGG | 0.21 | 0.27 | CGT |
|  | CGA | 0.11 | 0.09 | CGT |
|  | CGT | 0.08 | 0.03 | No change |
|  | CGC | 0.18 | 0.05 | CGT |
|  | CGG | 0.2 | 0.08 | CGT |
| Serine (Ser) | AGC | 0.24 | 0.2 | TCG |
|  | AGT | 0.15 | 0.19 | TCG |
|  | TCA | 0.15 | 0.25 | TCG |
|  | TCC | 0.22 | 0.14 | TCG |
|  | TCG | 0.05 | 0.05 | No change |
|  | TCT | 0.19 | 0.17 | TCG |
| Threonine (Thr) | ACA | 0.28 | 0.43 | ACG |
|  | ACC | 0.36 | 0.21 | ACG |
|  | ACG | 0.11 | 0.08 | No change |
|  | ACT | 0.25 | 0.29 | ACG |
| Valine (Val) | GTA | 0.12 | 0.22 | GTC |
|  | GTC | 0.24 | 0.2 | No change |
|  | GTG | 0.46 | 0.35 | GTC |
|  | GTT | 0.18 | 0.24 | GTC |
| Tyrosine (Tyr) | TAC | 0.56 | 0.47 | No change |
|  | TAT | 0.44 | 0.53 | TAC |
| Histidine (His) | CAC | 0.58 | 0.42 | No change |
|  | CAT | 0.42 | 0.58 | CAC |
| Isoleucine (Ile) | ATA | 0.17 | 0.37 | ATC |
|  | ATC | 0.47 | 0.27 | No change |
|  | ATT | 0.36 | 0.36 | ATC |
| Lysine (Lys) | AAA | 0.43 | 0.62 | AAG |
|  | AAG | 0.57 | 0.38 | No change |
| Leucine (Leu) | CTA | 0.07 | 0.17 | TTA |
|  | CTC | 0.2 | 0.15 | TTA |
|  | CTG | 0.4 | 0.19 | TTA |
|  | CTT | 0.13 | 0.21 | TTA |
|  | TTA | 0.08 | 0.1 | No change |
|  | TTG | 0.13 | 0.18 | TTA |

^a)^, Relative synonymous codon usage frequency was represented as fraction per amino acid (data adapted from reference [36])

**Table S2. Codon-reprogrammed sequences**

> PR8-PB2^rp^

AGCGAAAGCAGGTCAATTATATTCAATATGGAAAGAATAAAAGAACTAAGAAATCTAATGTCGCAGTCTCGCACCCGCGAGATACTCACAAAAACCACCGTGGACCATATGGCCATAATCAAGAAGTACACGTCGGGCCGTCAGGAGAAGAACCCGGCGTTACGTATGAAGTGGATGATGGCGATGAAGTACCCGATCACGGCGGACAAGCGTATCACGGAGATGATCCCGGAGCGTAACGAGCAGGGCCAGACGTTATGGTCGAAGATGAACGACGCGGGCTCGGACCGTGTCATGGTCTCGCCGTTAGCGGTCACGTGGTGGAACCGTAACGGCCCGATCACGAACACGGTCCACTACCCGAAGATCTACAAGACGTACTTTGAGCGTGTCGAGCGTTTAAAGCACGGCACGTTTGGCCCGGTCCACTTTCGTAACCAGGTCAAGATCCGTCGTCGTGTCGACATCAACCCGGGCCACGCGGACTTATCGGCGAAGGAGGCGCAGGACGTCATCATGGAGGTCGTCTTTCCGAACGAGGTCGGCGCGCGTATCTTAACGTCGGAGTCGCAGTTAACGATCACGAAGGAGAAGAAGGAGGAGTTACAGGACTGTAAGATCTCGCCGTTAATGGTCGCGTACATGTTAGAGCGTGAGTTAGTCCGTAAGACGCGTTTTTTACCGGTCGCGGGCGGCACGTCGTCGGTCTACATCGAGGTCTTACACTTAACGCAGGGCACGTGTTGGGAGCAGATGTACACGCCGGGCGGCGAGGTCCGTAACGACGACGTCGACCAGTCGTTAATCATCGCGGCGCGTAACATCGTCCGTCGTGCGGCGGTCTCGGCGGACCCGTTAGCGTCGTTATTAGAGATGTGTCACTCGACGCAGATCGGCGGCATCCGTATGGTCGACATCTTACGTCAGAACCCGACGGAGGAGCAGGCGGTCGACATCTGTAAGGCGGCGATGGGCTTACGTATCTCGTCGTCGTTTTCGTTTGGCGGCTTTACGTTTAAGCGTACGTCGGGCTCGTCGGTCAAGCGTGAGGAGGAGGTCTTAACGGGCAACTTACAGACGTTAAAGATCCGTGTCCACGAGGGCTACGAGGAGTTTACGATGGTCGGCCGTCGTGCGACGGCGATCTTACGTAAGGCGACGCGTCGTTTAATCCAGTTAATCGTCTCGGGCCGTGACGAGCAGTCGATCGCGGAGGCGATCATCGTCGCGATGGTCTTTTCGCAGGAGGACTGTATGATCAAGGCGGTCCGTGGCGACTTAAACTTTGTCAACCGTGCGAACCAGCGTTTAAACCCGATGCACCAGTTATTACGTCACTTTCAGAAGGACGCGAAGGTCTTATTTCAGAACTGGGGCGTCGAGCCGATCGACAACGTCATGGGCATGATCGGCATCTTACCGGACATGACGCCGTCGATCGAGATGTCGATGCGTGGCGTCCGTATCTCGAAGATGGGCGTCGACGAGTACTCGTCGACGGAGCGTGTCGTCGTCTCGATCGACCGTTTTTTACGTATCCGTGACCAGCGTGGCAACGTCTTATTATCGCCGGAGGAGGTCTCGGAGACGCAGGGCACGGAGAAGTTAACGATCACGTACTCGTCGTCGATGATGTGGGAGATCAACGGCCCGGAGTCGGTCTTAGTCAACACGTACCAGTGGATCATCCGTAACTGGGAGACGGTCAAGATCCAGTGGTCGCAGAACCCGACGATGTTATACAACAAGATGGAGTTTGAGCCGTTTCAGTCGTTAGTCCCGAAGGCGATCCGTGGCCAGTACTCGGGCTTTGTCCGTACGTTATTTCAGCAGATGCGTGACGTCTTAGGCACGTTTGACACGGCGCAGATCATCAAGTTATTACCGTTTGCGGCGGCGCCGCCGAAGCAGTCGCGTATGCAGTTTTCGTCGTTTACGGTCAACGTCCGTGGCTCGGGCATGCGTATCTTAGTCCGTGGCAACTCGCCGGTCTTTAACTACAACAAGGCGACGAAGCGTTTAACGGTCTTAGGCAAGGACGCGGGCACGTTAACGGAGGACCCGGACGAGGGCACGGCGGGCGTCGAGTCGGCGGTCTTACGTGGCTTTTTAATCTTAGGCAAGGAGGACAAGCGTTACGGCCCGGCGTTATCGATCAACGAGTTATCGAACTTAGCGAAGGGCGAGAAGGCGAACGTCTTAATCGGCCAGGGCGACGTGGTGTTGGTAATGAAACGGAAACGGGACTCTAGCATACTTACTGACAGCCAGACAGCGACCAAAAGAATTCGGATGGCCATCAATTAGTGTCGAATAGTTTAAAAACGACCTTGTTTCTACT

> PR8-HA^rp^

AGCAAAAGCAGGGGAAAATAAAAACAACCAAAATGAAGGCAAACCTATTAGTCTTATTATCGGCGTTAGCGGCGGCGGACGCGGACACGATCTGTATCGGCTACCACGCGAACAACTCGACGGACACGGTCGACACGGTCTTAGAGAAGAACGTCACGGTCACGCACTCGGTCAACTTATTAGAGGACTCGCACAACGGCAAGTTATGTCGTTTAAAGGGCATCGCGCCGTTACAGTTAGGCAAGTGTAACATCGCGGGCTGGTTATTAGGCAACCCGGAGTGTGACCCGTTATTACCGGTCCGTTCGTGGTCGTACATCGTCGAGACGCCGAACTCGGAGAACGGCATCTGTTACCCGGGCGACTTTATCGACTACGAGGAGTTACGTGAGCAGTTATCGTCGGTCTCGTCGTTTGAGCGTTTTGAGATCTTTCCGAAGGAGTCGTCGTGGCCGAACCACAACACGAACGGCGTCACGGCGGCGTGTTCGCACGAGGGCAAGTCGTCGTTTTACCGTAACTTATTATGGTTAACGGAGAAGGAGGGCTCGTACCCGAAGTTAAAGAACTCGTACGTCAACAAGAAGGGCAAGGAGGTCTTAGTCTTATGGGGCATCCACCACCCGCCGAACTCGAAGGAGCAGCAGAACATCTACCAGAACGAGAACGCGTACGTCTCGGTCGTCACGTCGAACTACAACCGTCGTTTTACGCCGGAGATCGCGGAGCGTCCGAAGGTCCGTGACCAGGCGGGCCGTATGAACTACTACTGGACGTTATTAAAGCCGGGCGACACGATCATCTTTGAGGCGAACGGCAACTTAATCGCGCCGATGTACGCGTTTGCGTTATCGCGTGGCTTTGGCTCGGGCATCATCACGTCGAACGCGTCGATGCACGAGTGTAACACGAAGTGTCAGACGCCGTTAGGCGCGATCAACTCGTCGTTACCGTACCAGAACATCCACCCGGTCACGATCGGCGAGTGTCCGAAGTACGTCCGTTCGGCGAAGTTACGTATGGTCACGGGCTTACGTAACACGCCGTCGATCCAGTCGCGTGGCTTATTTGGCGCGATCGCGGGCTTTATCGAGGGCGGCTGGACGGGCATGATCGACGGCTGGTACGGCTACCACCACCAGAACGAGCAGGGCTCGGGCTACGCGGCGGACCAGAAGTCGACGCAGAACGCGATCAACGGCATCACGAACAAGGTCAACACGGTCATCGAGAAGATGAACATCCAGTTTACGGCGGTCGGCAAGGAGTTTAACAAGTTAGAGAAGCGTATGGAGAACTTAAACAAGAAGGTCGACGACGGCTTTTTAGACATCTGGACGTACAACGCGGAGTTATTAGTCTTATTAGAGAACGAGCGTACGTTAGACTTTCACGACTCGAACGTCAAGAACTTATACGAGAAGGTCAAGTCGCAGTTAAAGAACAACGCGAAGGAGATCGGCAACGGCTGTTTTGAGTTTTACCACAAGTGTGACAACGAGTGTATGGAGTCGGTCCGTAACGGCACGTACGACTACCCGAAGTACTCGGAGGAGTCGAAGTTAAACCGTGAGAAGGTCGACGGCGTCAAGTTAGAGTCGATGGGCATCTACCAGATCTTAGCGATCTACTCGACGGTCGCGTCGTCGCTGGTGCTTTTGGTCTCCCTGGGGGCAATCAGTTTCTGGATGTGTTCTAATGGATCTTTGCAGTGCAGAATATGCATCTGAGATTAGAATTTCAGAAATATGAGGAAAAACACCCTTGTTTCTACT

>PR8-NP^rp^

AGCAAAAGCAGGGTAGATAATCACTCACTGAGTGACATCAAAATCATGGCGTCCCAAGGCACCAAACGGTCTTACGAACAGATGGAGACTGATGGAGAACGCCAGAACGCGACGGAGATCCGTGCGTCGGTCGGCAAGATGATCGGCGGCATCGGCCGTTTTTACATCCAGATGTGTACGGAGTTAAAGTTATCGGACTACGAGGGCCGTTTAATCCAGAACTCGTTAACGATCGAGCGTATGGTCTTATCGGCGTTTGACGAGCGTCGTAACAAGTACTTAGAGGAGCACCCGTCGGCGGGCAAGGACCCGAAGAAGACGGGCGGCCCGATCTACCGTCGTGTCAACGGCAAGTGGATGCGTGAGTTAATCTTATACGACAAGGAGGAGATCCGTCGTATCTGGCGTCAGGCGAACAACGGCGACGACGCGACGGCGGGCTTAACGCACATGATGATCTGGCACTCGAACTTAAACGACGCGACGTACCAGCGTACGCGTGCGTTAGTCCGTACGGGCATGGACCCGCGTATGTGTTCGTTAATGCAGGGCTCGACGTTACCGCGTCGTTCGGGCGCGGCGGGCGCGGCGGTCAAGGGCGTCGGCACGATGGTCATGGAGTTAGTCCGTATGATCAAGCGTGGCATCAACGACCGTAACTTTTGGCGTGGCGAGAACGGCCGTAAGACGCGTATCGCGTACGAGCGTATGTGTAACATCTTAAAGGGCAAGTTTCAGACGGCGGCGCAGAAGGCGATGATGGACCAGGTCCGTGAGTCGCGTAACCCGGGCAACGCGGAGTTTGAGGACTTAACGTTTTTAGCGCGTTCGGCGTTAATCTTACGTGGCTCGGTCGCGCACAAGTCGTGTTTACCGGCGTGTGTCTACGGCCCGGCGGTCGCGTCGGGCTACGACTTTGAGCGTGAGGGCTACTCGTTAGTCGGCATCGACCCGTTTCGTTTATTACAGAACTCGCAGGTCTACTCGTTAATCCGTCCGAACGAGAACCCGGCGCACAAGTCGCAGTTAGTCTGGATGGCGTGTCACTCGGCGGCGTTTGAGGACTTACGTGTCTTATCGTTTATCAAGGGCACGAAGGTCTTACCGCGTGGCAAGTTATCGACGCGTGGCGTCCAGATCGCGTCGAACGAGAACATGGAGACGATGGAGTCGTCGACGTTAGAGTTACGTTCGCGTTACTGGGCGATCCGTACGCGTTCGGGCGGCAACACGAACCAGCAGCGTGCGTCGGCGGGCCAGATCTCGATCCAGCCGACGTTTTCGGTCCAGCGTAACTTACCGTTTGACCGTACGACGATCATGGCGGCGTTTAACGGCAACACGGAGGGCCGTACGTCGGACATGCGTACGGAGATCATCCGTATGATGGAGTCGGCGCGTCCGGAGGACGTCTCGTTTCAGGGGCGGGGAGTCTTCGAGCTCTCGGACGAAAAGGCAGCGAGCCCGATCGTGCCTTCCTTTGACATGAGTAATGAAGGATCTTATTTCTTCGGAGACAATGCAGAGGAGTACGACAATTAAAGAAAAATACCCTTGTTTCTACT

>PR8-NA^rp^

AGCGAAAGCAGGGGTTTAAAATGAATCCAAATCAGAAAATAACGACGATCGGCTCGATCTGTTTAGTCGTCGGCTTAATCTCGTTAATCTTACAGATCGGCAACATCATCTCGATCTGGATCTCGCACTCGATCCAGACGGGCTCGCAGAACCACACGGGCATCTGTAACCAGAACATCATCACGTACAAGAACTCGACGTGGGTCAAGGACACGACGTCGGTCATCTTAACGGGCAACTCGTCGTTATGTCCGATCCGTGGCTGGGCGATCTACTCGAAGGACAACTCGATCCGTATCGGCTCGAAGGGCGACGTCTTTGTCATCCGTGAGCCGTTTATCTCGTGTTCGCACTTAGAGTGTCGTACGTTTTTTTTAACGCAGGGCGCGTTATTAAACGACAAGCACTCGAACGGCACGGTCAAGGACCGTTCGCCGTACCGTGCGTTAATGTCGTGTCCGGTCGGCGAGGCGCCGTCGCCGTACAACTCGCGTTTTGAGTCGGTCGCGTGGTCGGCGTCGGCGTGTCACGACGGCATGGGCTGGTTAACGATCGGCATCTCGGGCCCGGACAACGGCGCGGTCGCGGTCTTAAAGTACAACGGCATCATCACGGAGACGATCAAGTCGTGGCGTAAGAAGATCTTACGTACGCAGGAGTCGGAGTGTGCGTGTGTCAACGGCTCGTGTTTTACGATCATGACGGACGGCCCGTCGGACGGCTTAGCGTCGTACAAGATCTTTAAGATCGAGAAGGGCAAGGTCACGAAGTCGATCGAGTTAAACGCGCCGAACTCGCACTACGAGGAGTGTTCGTGTTACCCGGACACGGGCAAGGTCATGTGTGTCTGTCGTGACAACTGGCACGGCTCGAACCGTCCGTGGGTCTCGTTTGACCAGAACTTAGACTACCAGATCGGCTACATCTGTTCGGGCGTCTTTGGCGACAACCCGCGTCCGGAGGACGGCACGGGCTCGTGTGGCCCGGTCTACGTCGACGGCGCGAACGGCGTCAAGGGCTTTTCGTACCGTTACGGCAACGGCGTCTGGATCGGCCGTACGAAGTCGCACTCGTCGCGTCACGGCTTTGAGATGATCTGGGACCCGAACGGCTGGACGGAGACGGACTCGAAGTTTTCGGTCCGTCAGGACGTCGTCGCGATGACGGACTGGTCGGGCTACTCGGGCTCGTTTGTCCAGCACCCGGAGTTAACGGGCTTAGACTGTATGCGTCCGTGTTTTTGGGTCGAGTTAATCCGTGGCCGTCCGAAGGAGAAGACGATCTGGACGTCGGCGTCGTCGATCTCGTTTTGTGGCGTCAACTCGGACACGGTCGACTGGTCGTGGCCAGACGGTGCTGAGTTGCCATTCAGCATTGACAAGTAGTCTGTTCAAAAAACTCCTTGTTTCTACT

>PR8-NS^rp^

AGCAAAAGCAGGGTGACAAAGACATAATGGATCCAAACACTGTGTCAAGCTTTCAGGTAGATTGCTTTCTTTGGCATGTCCGCAAACGAGTTGCAGACCAAGAACTAGGTGATGCCCCATTCCTTGATCGGCTTCGCCGAGATCAGAAATCCCTAAGAGGAAGGGGCAGCACCCTCGGCTTAGACATCGAGACGGCGACGCGTGCGGGCAAGCAGATCGTCGAGCGTATCTTAAAGGAGGAGTCGGACGAGGCGTTAAAGATGACGATGGCGTCGGTCCCGGCGTCGCGTTACTTAACGGACATGACGTTAGAGGAGATGTCGCGTGACTGGTCGATGTTAATCCCGAAGCAGAAGGTCGCGGGCCCGTTATGTATCCGTATGGACCAGGCGATCATGGACAAGAACATCATCTTAAAGGCGAACTTTTCGGTCATCTTTGACCGTTTAGAGACGTTAATCTTATTACGTGCGTTTACGGAGGAGGGCGCGATCGTCGGCGAGATCTCACCATTGCCTTCTCTTCCAGGACATACTGCTGAGGATGTCAAAAATGCAGTTGGAGTCCTCATCGGGGGACTTGAATGGAATGATAACACAGTTCGAGTCTCTGAAACTCTACAGAGATTCGCTTGGAGAAGCAGTAATGAGAATGGGAGACCTCCACTCACTCCAAAACAGAAACGAGAAATGGCGGGAACAATTAGGTCAGAAGTTTGAAGAAATAAGATGGTTGATTGAAGAAGTGAGACACAAACTGAAGATAACAGAGAATAGTTTTGAGCAAATAACATTTATGCAAGCCTTACATCTATTGCTTGAAGTGGAGCAAGAGATAAGAACTTTCTCGTTTCAGCTTATTTAATAATAAAAAACACCCTTGTTTCTACT

>Vic4897-HA^rp^

AGCAAAAGCAGGGGAAAATAAAAGCAACAAAAATGAAGGCAATACTAGTCGTCATGTTATACACGTTTACGACGGCGAACGCGGACACGTTATGTATCGGCTACCACGCGAACAACTCGACGGACACGGTCGACACGGTCTTAGAGAAGAACGTCACGGTCACGCACTCGGTCAACTTATTAGAGGACAAGCACAACGGCAAGTTATGTAAGTTACGTGGCGTCGCGCCGTTACACTTAGGCCAGTGTAACATCGCGGGCTGGATCTTAGGCAACCCGGAGTGTGAGTCGTTATCGACGGCGCGTTCGTGGTCGTACATCGTCGAGACGTCGAACTCGGACAACGGCACGTGTTACCCGGGCGACTTTATCAACTACGAGGAGTTACGTGAGCAGTTATCGTCGGTCTCGTCGTTTGAGCGTTTTGAGATCTTTCCGAAGACGTCGTCGTGGCCGAACCACGACTCGGACAACGGCGTCACGGCGGCGTGTTCGCACGCGGGCGCGAAGTCGTTTTACAAGAACTTAATCTGGTTAGTCAAGAAGGGCAAGTCGTACCCGAAGATCAACCAGACGTACATCAACGACAAGGGCAAGGAGGTCTTAGTCTTATGGGGCATCCACCACCCGCCGACGATCACGGACCAGGAGTCGTTATACCAGAACGCGGACGCGTACGTCTTTGTCGGCACGTCGCGTTACTCGAAGAAGTTTAAGCCGGAGATCGCGGCGCGTCCGAAGGTCCGTGACCGTGCGGGCCGTATGAACTACTACTGGACGTTAGTCGAGCCGGGCGACAAGATCACGTTTGAGGCGACGGGCAACTTAGTCGCGCCGCGTTACGCGTTTACGATGGAGAAGGAGGCGGGCTCGGGCATCATCATCTCGGACACGCCGGTCCACGACTGTAACGCGACGTGTCAGACGCCGGAGGGCGCGATCAACACGTCGTTACCGTTTCAGAACGTCCACCCGATCACGATCGGCAAGTGTCCGAAGTACGTCCGTTCGACGAAGTTACGTTTAGCGACGGGCTTACGTAACGTCCCGTCGATCCAGTCGCGTGGCTTATTTGGCGCGATCGCGGGCTTTATCGAGGGCGGCTGGACGGGCATGGTCGACGGCTGGTACGGCTACCACCACCAGAACGACCAGGGCTCGGGCTACGCGGCGGACTTAAAGTCGACGCAGAACGCGATCGACAAGATCACGAACAAGGTCAACTCGGTCATCGAGAAGATGAACACGCAGTTTACGGCGGTCGGCAAGGAGTTTAACCACTTAGAGAAGCGTATCGAGAACTTAAACAAGAAGGTCGACGACGGCTTTTTAGACGTCTGGACGTACAACGCGGAGTTATTAGTCTTATTAGAGAACGAGCGTACGTTAGACTACCACGACTCGAACGTCAAGAACTTATACGAGAAGGTCCGTCACCAGTTAAAGAACAACGCGAAGGAGATCGGCAACGGCTGTTTTGAGTTTTACCACAAGTGTGACAACACGTGTATGGAGTCGGTCAAGAACGGCACGTACGACTACCCGAAGTACTCGGAGGAGGCGAAGTTAAACCGTGAGAAGATCGACGGCGTCAAGTTAGACTCGACGCGTATCTACCAGATCTTAGCGATCTACTCGACGGTCGCGTCGTCGTTGGTACTGGTAGTCTCCCTGGGGGCAATCAGCTTCTGGATGTGCTCTAATGGGTCTCTACAGTGTAGAATATGTATTTAACATTAGAATTTCAGAATCATGAGAAAAAACACCCTTGTTTCTACT

>Vic4897-NA^rp^

AGCAAAAGCAGGAGTTTAAAATGAATCCAAACCAAAAGATAATCACGATCGGCTCGATCTGTATGACGATCGGCACGGCGAACTTAATCTTACAGATCGGCAACATCATCTCGATCTGGGTCTCGCACTCGATCCAGATCGGCAACCAGTCGCAGATCGAGACGTGTGACAAGTCGGTCATCACGTACGAGAACAACACGTGGGTCAACCAGACGTTTGTCAACATCTCGAACACGAACTCGGCGGCGCGTCAGTCGGTCGCGTCGGTCAAGTTAGCGGGCAACTCGTCGTTATGTCCGGTCTCGGGCTGGGCGATCTACTCGAAGGACAACTCGGTCCGTATCGGCTCGAAGGGCGACGTCTTTGTCATCCGTGAGCCGTTTATCTCGTGTTCGCCGTTAGAGTGTCGTACGTTTTTTTTAACGCAGGGCGCGTTATTAAACGACAAGCACTCGAACGGCACGATCAAGGACCGTTCGCCGTACCGTACGTTAATGTCGTGTCCGATCGGCGAGGTCCCGTCGCCGTACAACTCGCGTTTTGAGTCGGTCGCGTGGTCGGCGTCGGCGTGTCACGACGGCACGAACTGGTTAACGATCGGCATCTCGGGCCCGGACTCGGGCGCGGTCGCGGTCTTAAAGTACAACGGCATCATCACGGACACGATCAAGTCGTGGCGTAACAAGATCTTACGTACGCAGGAGTCGGAGTGTGCGTGTGTCAACGGCTCGTGTTTTACGATCATGACGGACGGCCCGTCGGACGGCCAGGCGTCGTACAAGATCTTTCGTATCGAGAAGGGCAAGATCATCAAGTCGGTCGAGATGAAGGCGCCGAACTACCACTACGAGGAGTGTTCGTGTTACCCGGACTCGTCGGAGATCACGTGTGTCTGTCGTGACAACTGGCACGGCTCGAACCGTCCGTGGGTCTCGTTTAACCAGAACTTAGAGTACCAGATGGGCTACATCTGTTCGGGCGTCTTTGGCGACAACCCGCGTCCGAACGACAAGACGGGCTCGTGTGGCCCGGTCTCGTCGAACGGCGCGAACGGCGTCAAGGGCTTTTCGTTTAAGTACGGCAACGGCGTCTGGATCGGCCGTACGAAGTCGATCTCGTCGCGTAAGGGCTTTGAGATGATCTGGGACCCGAACGGCTGGACGGAGACGGACAACAAGTTTTCGAAGAAGCAGGACATCGTCGGCATCAACGAGTGGTCGGGCTACTCGGGCTCGTTTGTCCAGCACCCGGAGTTAACGGGCTTAAACTGTATCCGTCCGTGTTTTTGGGTCGAGTTAATCCGTGGCCGTCCGGAGGAGAACACGATCTGGACGTCGGGCTCGTCGATCTCGTTTTGTGGCGTCGACTCGGACATCATGGGCTGGTCGTGGCCAGACGGTGCTGAGTTGCCATTCACCATTGACAATTAATTTGTTCAAAAAAACTCCTTGTTTCTACT

**Table S3. Characteristics of reprogrammed HA and NA of A/Victoria/4897/2022 (Vic4897)**

| Gene segment | Type | Substitutions | CAI | | C+G% | No. CpGs | No. UpAs |
| --- | --- | --- | --- | --- | --- | --- | --- |
|  |  |  | Relative to Homo sapiens^a)^ | Relative to Influenza A virus^b)^ |  |  |  |
| Vic4897 HA | Wildtype | - | 0.731 | 0.811 | 40.83 | 28 | 100 |
|  | Reprogrammed | **449** | **0.648** | **0.486** | **53.71** | **217** | **96** |
| Vic4897 NA | Wildtype | - | 0.744 | 0.8 | 41.19 | 22 | 83 |
|  | Reprogrammed | **393** | **0.633** | **0.461** | **55.72** | **185** | **47** |

^a)^, The codon adaptation indexes were calculated to quantify the similarities in codon usage between them and the Homo sapiens genome reference by using CAIcal (https://ppuigbo.me/programs/CAIcal/).

^b)^, The codon adaptation indexes were calculated to quantify the similarities in codon usage between them and the Influenza A virus genome reference.

**Table S4.** **Primers used to quantify mRNA or vRNA expression of viral genes and mRNA expression of antiviral markers**

| Gene | Reverse transcript Primer (5′-3′) | Quantitative PCR primer | |
| --- | --- | --- | --- |
|  |  | Forward Primer (5′-3′) | Reverse Primer (5′-3′) |
| For mRNA of viral genes | | | |
| PB2 | CCAGATCGTTCGAGTCGTTTTTTTTTTTTTTTTTAAACTATTCGA | GMGACGTGGTGTTGGTAATG | CCAGATCGTTCGAGTCGT |
| PB1 | CCAGATCGTTCGAGTCGTTTTTTTTTTTTTTTTTCATGAAGGACA | GTTTCCAGAGCCCGAATTGA | CCAGATCGTTCGAGTCGT |
| PA | CCAGATCGTTCGAGTCGTTTTTTTTTTTTTTTTTGGACAGTATGG | GCTCTTAGGGACAACCTTGAA | CCAGATCGTTCGAGTCGT |
| HA | CCAGATCGTTCGAGTCGTTTTTTTTTTTTTTTTTCCTCATATTTC | AGATYYTRGCGATCTACTCRAC | CCAGATCGTTCGAGTCGT |
| NP | CCAGATCGTTCGAGTCGTTTTTTTTTTTTTTTTTCTTTAATTGTC | TTCGAGCTCTCGGACGAA | CCAGATCGTTCGAGTCGT |
| NA | CCAGATCGTTCGAGTCGTTTTTTTTTTTTTTTTTGAACAGACTAC | TATGMGKCCGTGYTTYTG | CCAGATCGTTCGAGTCGT |
| M | CCAGATCGTTCGAGTCGTTTTTTTTTTTTTTTTTACTCCAGCTCT | GCCAAAGTCTATGAGGGAAGAA | CCAGATCGTTCGAGTCGT |
| NS | CCAGATCGTTCGAGTCGTTTTTTTTTTTTTTTTTATTATTAAATA | GCCTTCTCTTCCAGGACATAC | CCAGATCGTTCGAGTCGT |
| Canine GAPDH | TTTTTTTTTTTTTTTTTTTTTTTVN^a)^ | GATGCTGGTGCTGAGTATGT | CAGAAGGAGCAGAGATGATGAC |
| For vRNA of viral genes | | | |
| PB2 | GGCCGTCATGGTGGCGAATGMGACGTGGTGTTGGTAATG | GGCCGTCATGGTGGCGAAT | ATGGCCATCCGAATTCTTT |
| PB1 | GGCCGTCATGGTGGCGAATAGTCAGCGGACATGAGTATTG | GGCCGTCATGGTGGCGAAT | GAACAACTGAAGGGCCATTTG |
| PA | GGCCGTCATGGTGGCGAATGAGCTCGATGAGATTGGAGAAG | GGCCGTCATGGTGGCGAAT | GTGAGACACCTCTGATGTGAAA |
| HA | GGCCGTCATGGTGGCGAATAGATYYTRGCGATCTACTCRAC | GGCCGTCATGGTGGCGAAT | CTGCACTGCAAAGATCCATTAG |
| NP | GGCCGTCATGGTGGCGAATTTCGAGCTCTCGGACGAA | GGCCGTCATGGTGGCGAAT | GTCTCCGAAGAAATAAGATCCTTCA |
| NA | GGCCGTCATGGTGGCGAATTATGMGKCCGTGYTTYTG | GGCCGTCATGGTGGCGAAT | TGCTGAATGGCAACTCAG |
| M | GGCCGTCATGGTGGCGAATGACCAATCCTGTCACCTCTGAC | GGCCGTCATGGTGGCGAAT | AGGGCATTYTGGACAAAKCGTCTA |
| NS | GGCCGTCATGGTGGCGAATGCCTTCTCTTCCAGGACATAC | GGCCGTCATGGTGGCGAAT | GGAGGTCTCCCATTCTCATTAC |
| For antiviral markers | | | |
| IFN-α | TTTTTTTTTTTTTTTTTTTTTTTVN^a)^ | AATTCTGCACCGAACTCTACC | ATGGAGTCCGCATTCATCAG |
| IFN-β | TTTTTTTTTTTTTTTTTTTTTTTVN^a^ | GGCAATTGAATGGGAGGCT | GGCGTCCTCCTTCTGGAACT |
| IFN-λ | TTTTTTTTTTTTTTTTTTTTTTTVN^a)^ | CGTGGTGCTGGTGACTTT | CTCCTGTGGTGACAGAGATTTG |
| ISG15 | TTTTTTTTTTTTTTTTTTTTTTTVN^a)^ | CGCAGATCACCCAGAAGATCG | TTCGTCGCATTTGTCCACCA |
| hGAPDH | TTTTTTTTTTTTTTTTTTTTTTTVN^a)^ | TCAAGGCTGAGAACGGGAAG | CGCCCCACTTGATTTTGGAG |

^a)^, Oligo(dT)23VN: V = A, C, or G; N = A, T, C, or G.

**Figure S1. Codon reprogramming attenuates a contemporary H1N1 strain.**

(A) Schematic representation of the recombinant Vic4897^rp^ genome, incorporating reprogrammed PB2, NP, and NS segments from PR8^rp^, reprogrammed HA and NA segments from Vic4897, and wild-type PB1, PA, and M segments from PR8. Numbers indicate the start and end positions of the reprogrammed regions within each segment. Blue bars represent PR8-derived segments, and coral bars represent Vic4897-derived segments. (B) Plaque morphology of wild-type Vic4897 and Vic4897^rp^ in MDCK cells, visualized by immunostaining at 4 days post-infection. Multi-cycle growth kinetics of Vic4897 and Vic4897^rp^ in embryonated chicken eggs (C), MDCK cells (D), and A549 cells (E) following infection with 100 PFU per egg or at an MOI of 0.01 for cell cultures. Viral titers were measured at the indicated times by plaque assay. Data are presented as mean ± SEM of three biological replicates. ***, *p* < 0.001; ****, *p* < 0.0001.


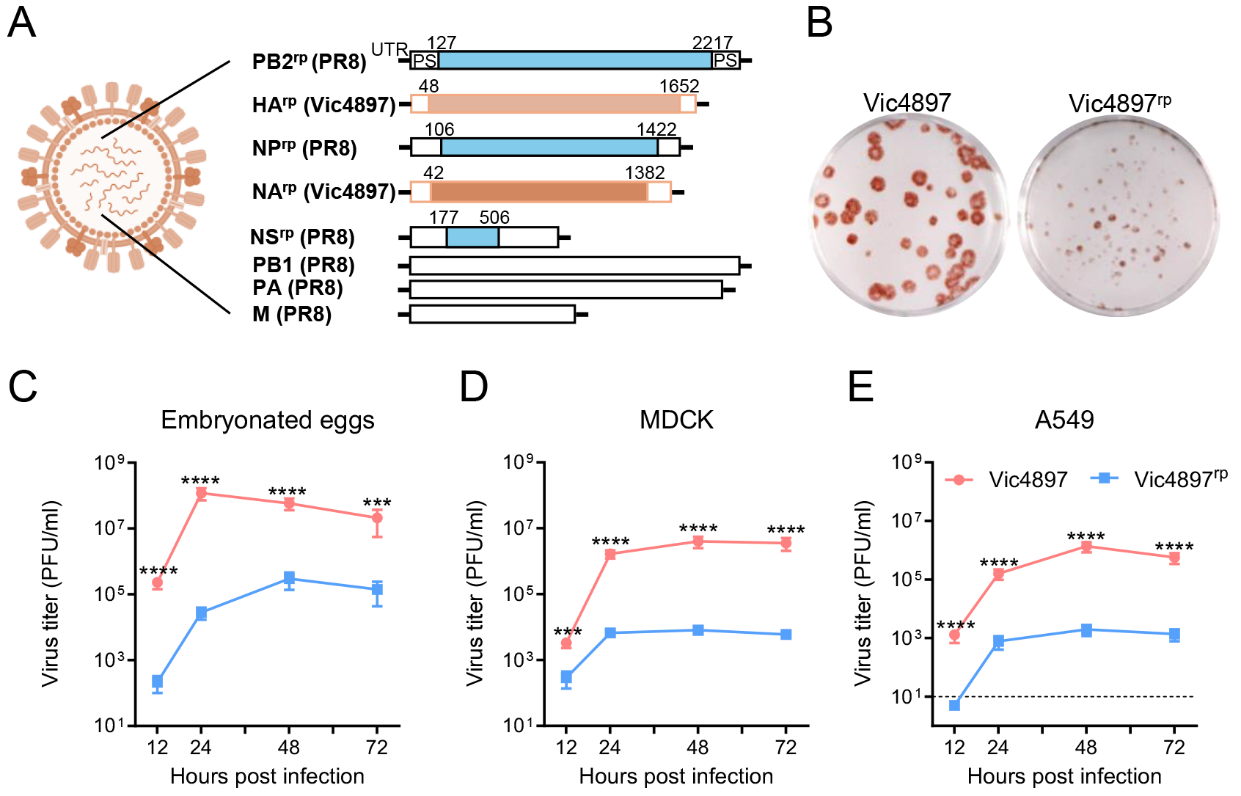


**Figure S2. Flow cytometric analysis of cytokine production in CD4⁺ T cells (related to Figure 5).**

(A, B) Representative flow cytometry plots showing IFN-γ^+^ (A) and IL-4^+^ (B) CD4^+^ T cells from mice immunized with PR8^rp^ (10^1^ or 10^4^ PFU) or PBS (NC) and stimulated *ex vivo* with inactivated PR8, H1N1pdm, H3N2, or NP antigens.


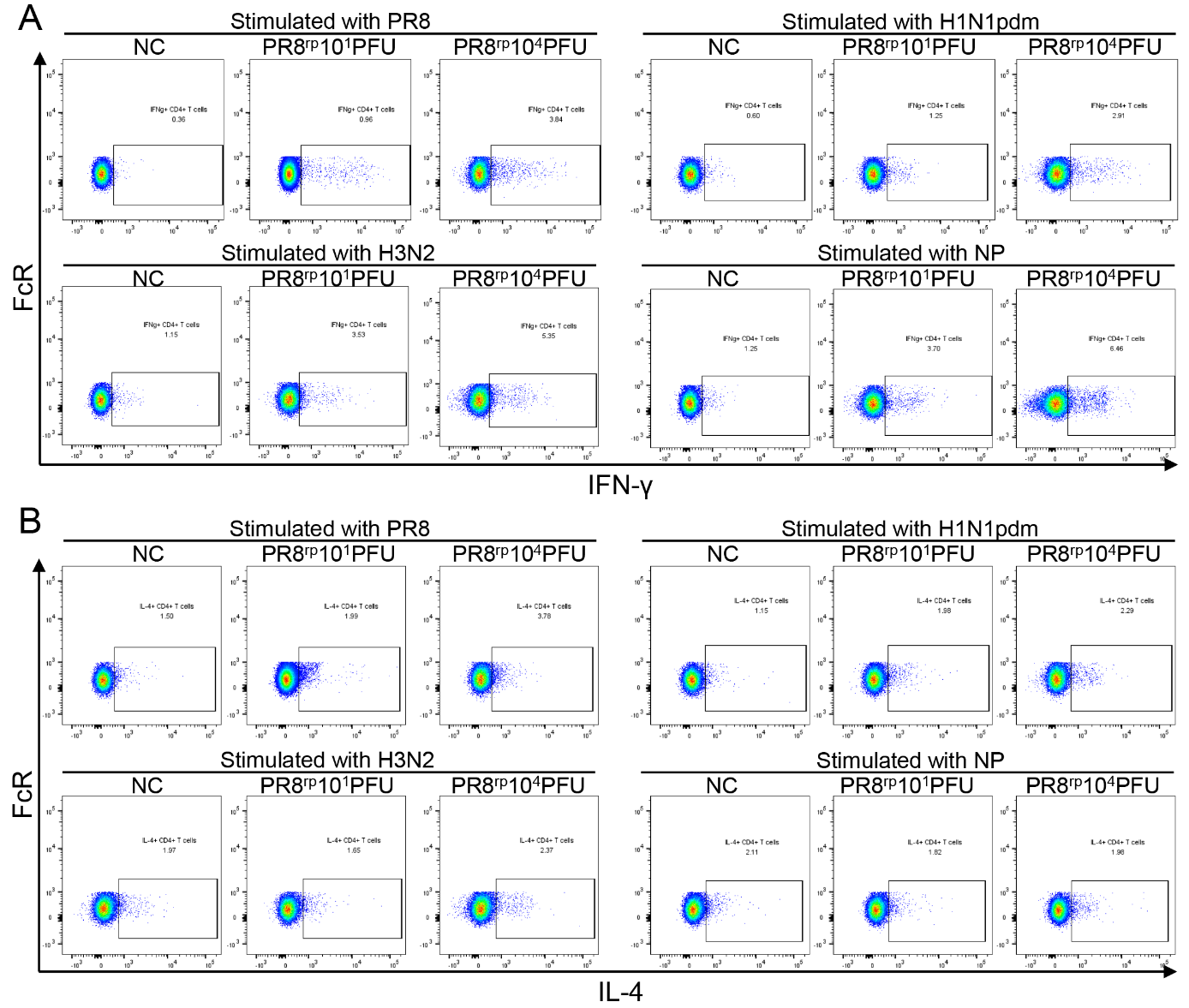


**Figure S3. Flow cytometric analysis of cytokine production in CD8⁺ T cells (related to Figure 5).**

(A, B) Representative flow cytometry plots showing IFN-γ^+^ (A) and IL-4^+^ (B) CD8^+^ T cells from mice immunized with PR8^rp^ (10^1^ or 10^4^ PFU) or PBS (NC) and stimulated *ex vivo* with inactivated PR8, H1N1pdm, H3N2, or NP antigens.


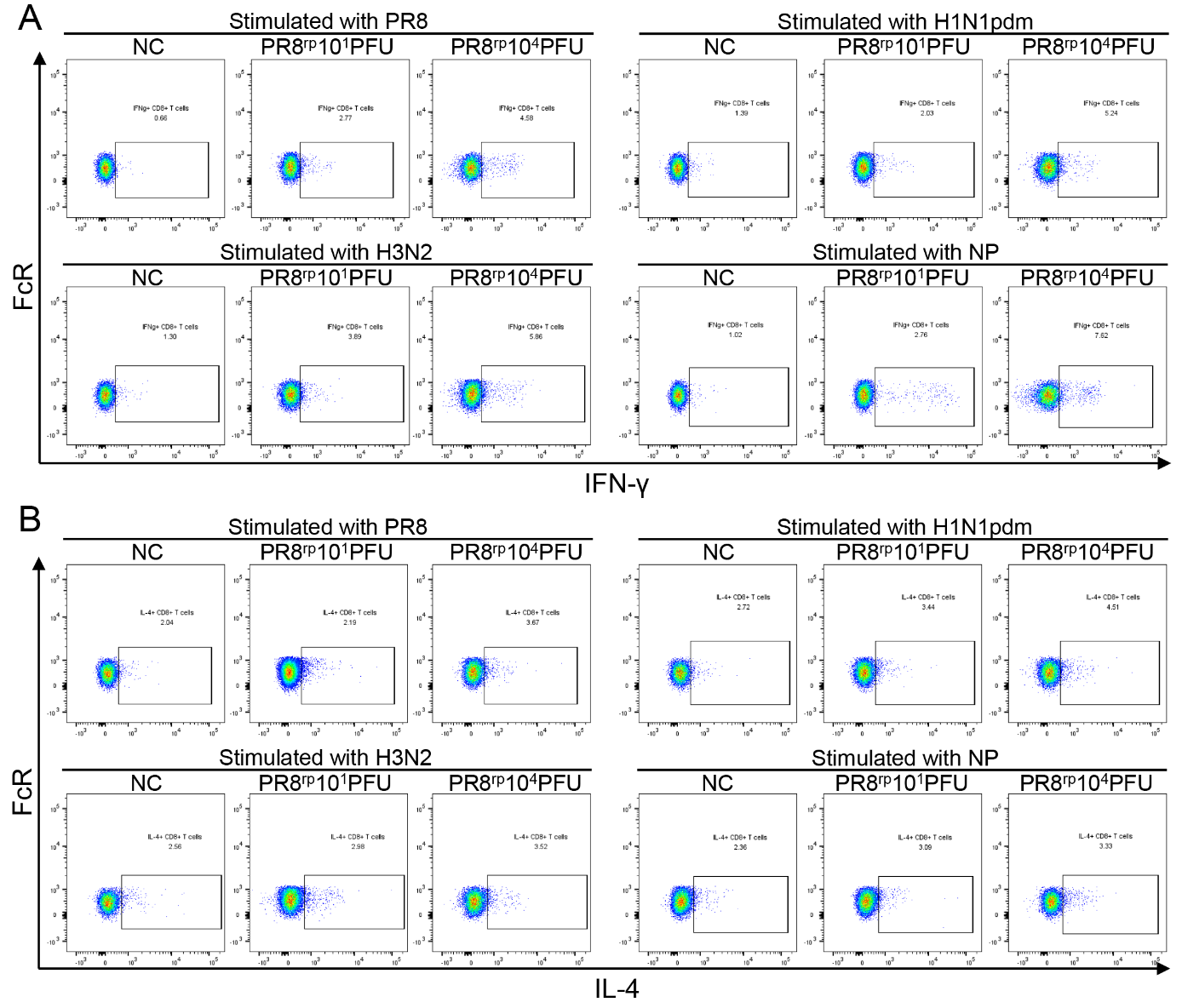

Supplement: Supplementary file 1 — Supporting Information [file ADVS-13-e16448-s001.docx]
